# Heme peroxidase HPX-2 protects *Caenorhabditis elegans* from pathogens

**DOI:** 10.1371/journal.pgen.1007944

**Published:** 2019-01-29

**Authors:** Yi Liu, Karan Gautam Kaval, Ambro van Hoof, Danielle A. Garsin

**Affiliations:** 1 Department of Microbiology and Molecular Genetics, The University of Texas Health Science Center at Houston, Houston TX, United States of America; 2 MD Anderson Cancer Center UTHealth Graduate School of Biomedical Sciences, Houston TX, United States of America; 3 The UT Center for Antimicrobial Resistance and Microbial Genomics, The University of Texas Health Science Center at Houston, Houston, TX, United States of America; Oregon Health and Science University, UNITED STATES

## Abstract

Heme-containing peroxidases are important components of innate immunity. Many of them functionally associate with NADPH oxidase (NOX)/dual oxidase (DUOX) enzymes by using the hydrogen peroxide they generate in downstream reactions. *Caenorhabditis elegans* encodes for several heme peroxidases, and in a previous study we identified the ShkT-containing peroxidase, SKPO-1, as necessary for pathogen resistance. Here, we demonstrated that another peroxidase, HPX-2 (**H**eme-**P**ero**X**idase 2), is required for resistance against some, but not all pathogens. Tissue specific RNA interference (RNAi) revealed that HPX-2 functionally localizes to the hypodermis of the worm. In congruence with this observation, *hpx-2* mutant animals possessed a weaker cuticle structure, indicated by higher permeability to a DNA dye, but exhibited no obvious morphological defects. In addition, fluorescent labeling of HPX-2 revealed its expression in the pharynx, an organ in which BLI-3 is also present. Interestingly, loss of HPX-2 increased intestinal colonization of *E*. *faecalis*, suggesting its role in the pharynx may limit intestinal colonization. Moreover, disruption of a catalytic residue in the peroxidase domain of HPX-2 resulted in decreased survival on *E*. *faecalis*, indicating its peroxidase activity is required for pathogen resistance. Finally, RNA-seq analysis of an *hpx-2* mutant revealed changes in genes encoding for cuticle structural components under the non-pathogenic conditions. Under pathogenic conditions, genes involved in infection response were differentially regulated to a greater degree, likely due to increased microbial burden. In conclusion, the characterization of the heme-peroxidase, HPX-2, revealed that it contributes to *C*. *elegans* pathogen resistance through a role in generating cuticle material in the hypodermis and pharynx.

## Author summary

Reactive oxygen species (ROS) production by the host tissues is one of the first lines of defense when microbial infection occurs. ROS has been shown to be involved in multiple protective pathways in innate immunity. However, given the complexity of mammalian systems, the exact manner in which ROS are used for host defense remains incompletely understood. In this study, we use *Caenorhabditis elegans* as a simplified model system to decipher the protective functions of ROS in innate immunity. We describe a peroxidase, HPX-2, that protects *C*. *elegans* from multiple infectious microbes by strengthening barrier tissue. This finding brings insight into the mechanisms by which peroxidases utilizes ROS to contribute to innate immunity. With infectious diseases being one of the most important causes of morbidity and mortality around the world, understanding ROS production and its function in pathogen resistance will provide us with important information in developing new therapies against pathogens.

## Introduction

When exposed to pathogen, production of Reactive Oxygen Species (ROS) is one of the first innate immune responses initiated by the host. ROS can exert immune-protective effects by acting directly as an antimicrobial, serving as a signal to activate downstream responses, and/or contributing to the generation of physical barriers (Reviewed by [1, 2]). There are two major groups of enzymes involved in this process–the ROS producing NADPH oxidases/Dual-oxidases (NOX/DUOX) and the ROS utilizing, heme-containing peroxidases (Reviewed by [1, 2]). In many cases, these enzymes are functionally, and sometimes physically linked to contribute to innate immunity against pathogen. The most canonical case studied in mammalian systems is that of myeloperoxidase (MPO), which resides in the phagolysosome of macrophages and neutrophils and utilizes the superoxide produced by NOX2 to generate the powerful oxidant HOCl during the oxidative burst that occurs as a result of microbial engulfment (Reviewed by [3]). Additionally, it has been shown that lactoperoxidase (LPO), coupled with DUOX2, is responsible for the generation of the antibiotic oxidant hypothiocyanite (OSCN-) from H_2_O_2_ and thiocyanate (SCN-) on mucosal surfaces [4–6]. These responses are not limited to animals; plants also employ peroxidases and NADPH oxidases as part of their antimicrobial arsenal (Reviewed in [7]).

In *C*. *elegans*, the one functional NOX/DUOX encoded by the genome is BLI-3, a dual oxidase that produces H_2_O_2_ when animals are exposed to pathogen [8–11]. Like all dual oxidases, BLI-3 consists of an NADPH oxidase domain and a heme peroxidase domain [10]. The production of H_2_O_2_ from the NADPH oxidase domain of BLI-3 is required for pathogen resistance and point mutants that affect this domain significantly increase pathogen susceptibility [8, 12]. In addition to its role during infection, BLI-3 is crucial for cuticle development. The peroxidase domain uses the H_2_O_2_ produced by the NADPH oxidase domain to cross-link collagen proteins [10]. Another heme-binding peroxidase, MLT-7, additionally contributes to this process [13]. Mutations in the peroxidase domains of BLI-3 and MLT-7 that impair activity result in a blistered phenotype. However, these mutations do not affect pathogen susceptibility [14].

Since peroxidases in other systems do play a role in innate immunity, we hypothesized that one or more of the peroxidases encoded in the *C*. *elegans* genome would affect pathogen resistance. Indeed, previous studies in our lab showed that a ShkT domain containing peroxidase, which we named SKPO-1, is required for resistance to *E*. *faecalis* [14]. A *skpo-1* mutant was more susceptible to *E*. *faecalis* compared to the N2 reference strain. In addition to pathogen sensitivity, the *skpo-1* mutant also had an incomplete penetrance of dumpy phenotype, suggesting a potential role in the process of cuticle bio-generation. Interestingly, SKPO-1 localized to the hypodermis of the worm, a tissue where BLI-3 also is present, indicating a potential physical interaction between the two [14].

Here, we characterized the immune-protective peroxidase, F09F3.5, which we named HPX-2, for **H**eme **P**eroxidase 2. Using a combination of RNA interference (RNAi) and CRISPR-Cas9 mediated mutation, we demonstrated that HPX-2 is important for protecting *C*. *elegans* against some, but not all, pathogens. Through fluorescent-tagging and tissue-specific RNAi, we demonstrated that HPX-2 is present in the pharynx and the hypodermis of the worms. Despite low expression of its gene at the organismal level, HPX-2 functions in these two tissues and contributes to cuticle integrity; *hpx-2* mutants exhibited higher intestinal colonization by pathogens with thick cell walls and possessed weaker cuticles, more penetrant to dye. We also examined animals with a point mutation at the catalytic site of HPX-2 peroxidase domain and discovered that peroxidase activity is partly required for the protective function of HPX-2. Lastly, a transcriptomic analysis of the *hpx-2* mutant revealed an altered immune response when exposed to *E*. *faecalis*. In summary, the results of these experiments demonstrated that HPX-2 is an immuno-protective peroxidase likely functioning in the development of cuticle barrier tissue associated with the hypodermis and pharynx.

## Results

### HPX-2 protects against many, but not all, pathogens

**Fig 1:**
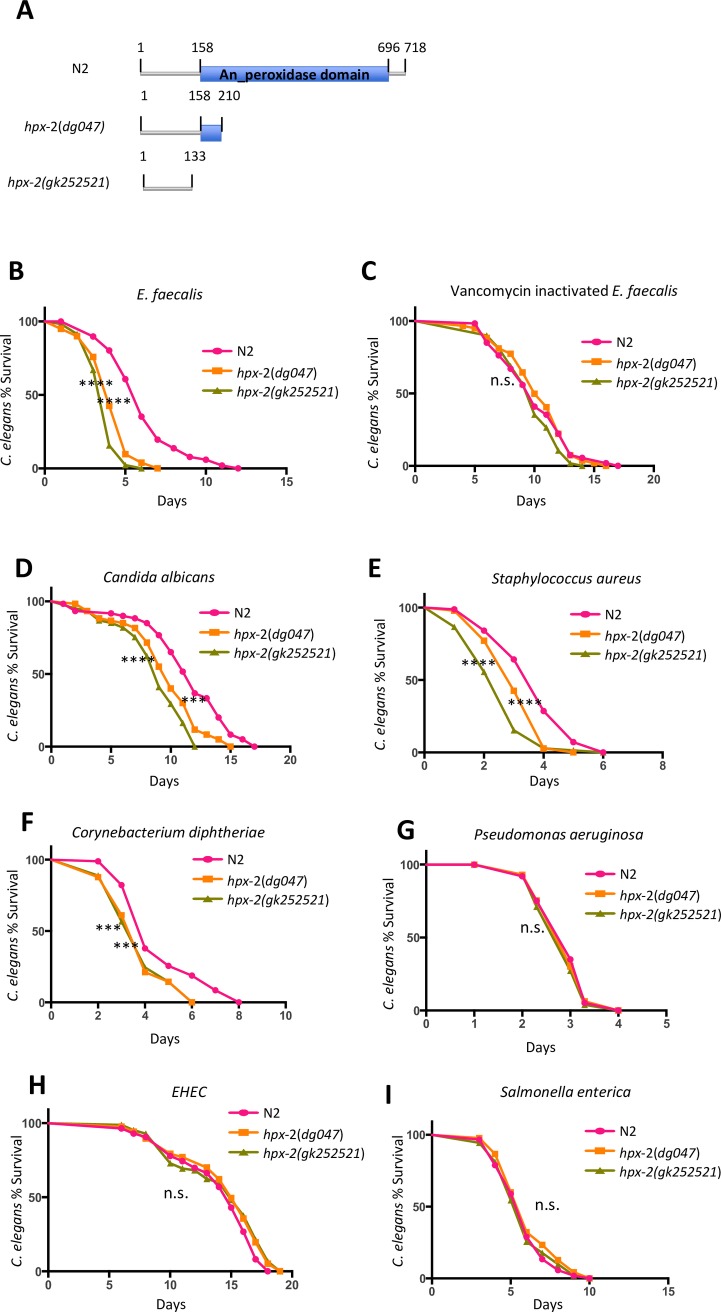
HPX-2 contributes to *C*. *elegans* resistance to some pathogens. (A) Schematic representation of the predicted HPX-2 translated products in N2, *hpx-2(dg047)*, and *hpx-2 (gk252521)*. (B) Survival of N2 and *hpx-2* mutants on live *Enterococcus faecalis* OG1RF. (C) Survival of N2 and *hpx-2* mutants on vancomycin inactivated *Enterococcus faecalis* OG1RF. Survival of N2 and *hpx-2* mutants on (D) *Candida albicans* SC5314, (E) *Staphylococcus aureus* NCTC8325, (F) *Corynebacterium diphtheriae* NCTC12129, *(*G) *Pseudomonas aeruginosa* PA14, (H) *Escherichia coli* O157:H7 Sakai (EHEC), and (I) *Salmonella enterica* SL1344. Results from one representative experiment with an n of approximately 90 worms for each condition are shown. Median survival and P-values are listed in S8 Table as experiment No.1 along with those from additional biological replicates.

Using RNAi, knock-down of *hpx-2* resulted in increased sensitivity of *C*. *elegans* to *E*. *faecalis* infection (S1 Fig), as previously reported [14]. To further study the function of HPX-2, we generated a truncated mutant allele (*hpx-2(dg047)*) by CRISPR-Cas9 mediated gene editing. *hpx-2(dg047)* is missing most of the predicted peroxidase domain and is a predicted loss-of-function mutant. We additionally obtained a nonsense mutant allele, *hpx-2(gk252521)*, from the *Caenorhabditis* Genetics Center that is predicted to produce a protein lacking the entire peroxidase domain (Fig 1A). The mutants were tested for general defects in fitness by measuring their longevity on live (S2A Fig) and heat-killed *E*. *coli* OP50 (S2B Fig). While both mutants showed slight longevity defects compared with the wild type reference strain, N2, on live *E*. *coli*, there was no significant difference on heat-killed *E*. *coli*. The minor longevity defect of the mutants on live *E*. *coli* OP50 might result from the slight pathogenicity of OP50 towards worms as they age [15]. Overall, these data suggest that the loss of HPX-2 does not dramatically affect the fitness of the worms.

We next tested the susceptibility of both *hpx-2* mutants to *E*. *faecalis*. In agreement with the RNAi experiment, both mutants showed significantly decreased survival on this species of bacteria compared to N2 (Fig 1B). Furthermore, the difference disappeared when worms were exposed to *E*. *faecalis* inactivated by vancomycin treatment, suggesting live bacteria were required for killing, and again indicating that the animals do not have a general fitness defect (Fig 1C). To test if the pathogen susceptibility of the *hpx-2* mutants is pathogen-specific, we exposed them to multiple human pathogens including the fungal pathogen *Candida albicans* SC5314 (Fig 1D), Gram-positive pathogens *Staphylococcus aureus* NCTC8325 (Fig 1E) and *Corynebacterium diphtheriae* NCTC12129 (Fig 1F), and finally Gram-negative pathogens *Pseudomonas aeruginosa* PA14 (Fig 1G), *Escherichia coli* O157:H7 Sakai (EHEC; Fig 1H), and *Salmonella enterica* SL1344 (Fig 1I). Interestingly, *hpx-2* mutants showed susceptibility to all the microbial pathogens tested except for the Gram-negative bacteria, raising the possibility that differences in microbial cell wall structure might impact the phenotype observed with loss of *hpx-2* (see Discussion).

**Fig 2:**
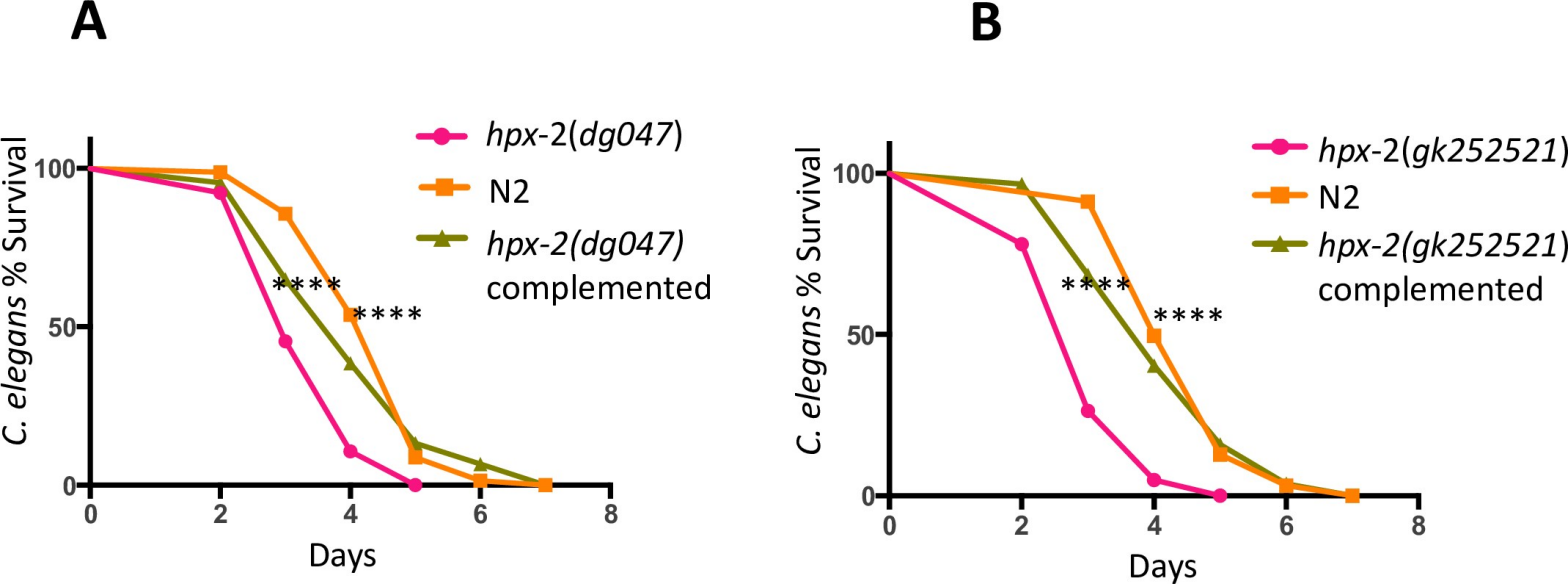
Genomic *hpx-2* complementation rescues the susceptibility to *E*. *faecalis*. (A) Survival of N2, *hpx-2(dg047)* mutant, and its complemented strain on *E*. *faecalis*. (B) Survival of N2, *hpx-2(gk252521)* mutant, and its complemented strain on *E*. *faecalis*. The *hpx-2* mutants are set as the control comparison. Results from one representative experiment with an n of approximately 90 worms for each condition are shown. Median survival and P-values are listed in S8 Table as experiment No.1 along with those from additional biological replicates.

To verify that HPX-2 is required for pathogen resistance, we complemented the mutant strains with DNA containing the *hpx-2* gene and subjected the animals to *E*. *faecalis* exposure. When exposed to *E*. *faecalis*, the complemented strains showed a significant increase in pathogen tolerance compared to the parental strains, reaching a level of resistance similar to N2 (Fig 2A and 2B). These data collectively suggest that HPX-2 is required for resistance to many, but not all, pathogens capable of infecting *C*. *elegans*.

### HPX-2 is present in the hypodermis and pharynx

**Fig 3:**
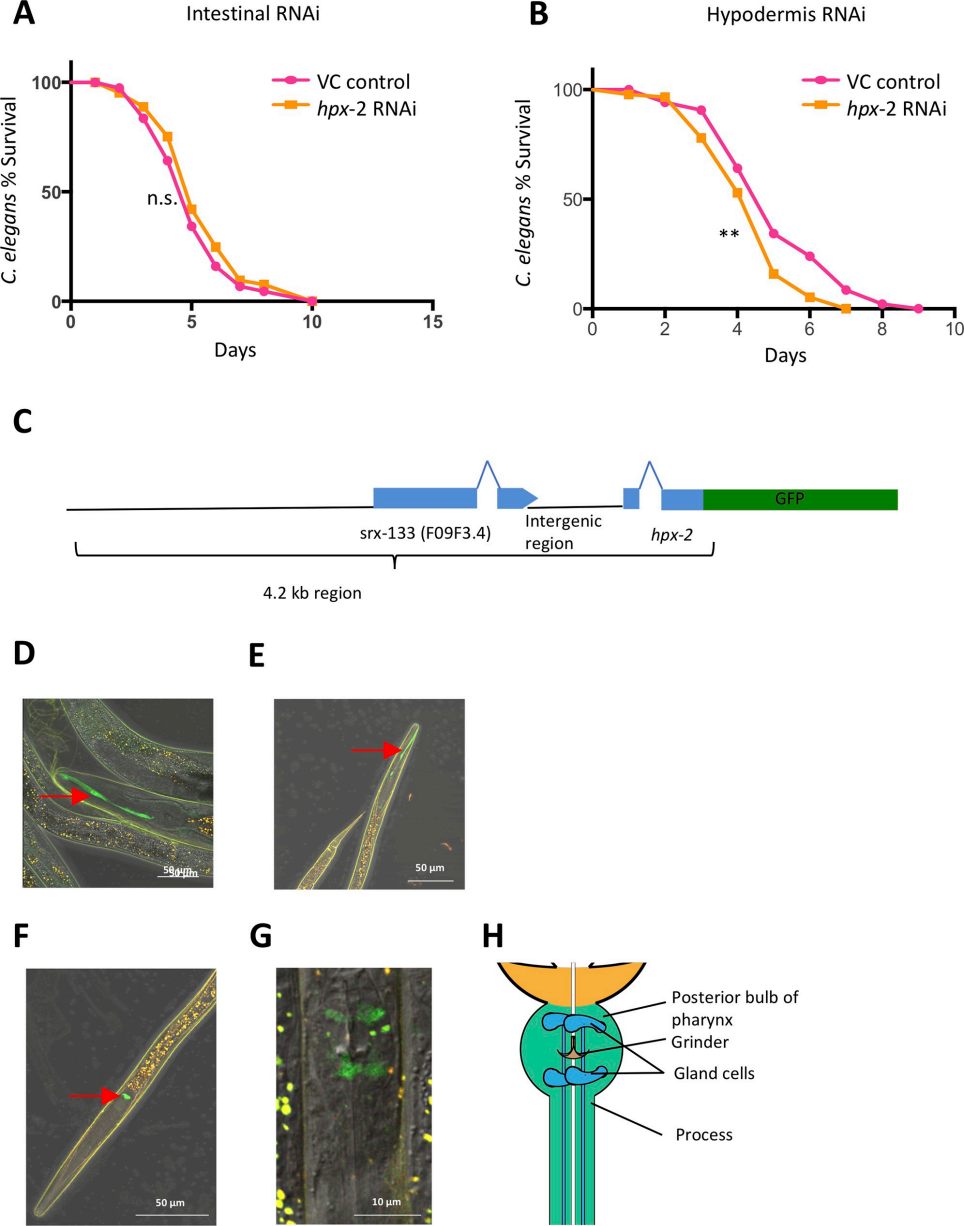
*hpx-2* is expressed in the hypodermis and pharynx. (A) Survival of the intestinal RNAi strain on *E*. *faecalis* following exposure to *hpx-2* or VC control RNAi. (B) Survival of the hypodermal RNAi strain on *E*. *faecalis* following exposure to *hpx-2* or VC control RNAi. Representative results from single experiments with an n of approximately 90 worms for each condition are shown. Median survival and P-values along with additional biological replicates are listed in S8 Table as experiment No.1. (C) Illustration of upstream region and the first two exons of the *hpx-2* gene that are fused to *gfp*. The expression pattern of *hpx-2*::*gfp* under the confocal microscope in young-adult worms (D)and dauer worms (E-G). Red arrow heads indicate the pharyngeal expression of *hpx-2* in either the processes (D and E) or the gland cells (F and G). (G) Higher magnification of the terminal bulb showing the pharyngeal expression pattern of *hpx-2*::*gfp*. (H) Diagram of the relevant features of the terminal bulb imaged in G. Note that D-G are mergers of images taken using the DIC, FITC and TRITC filters. Background autofluorescence is detected by both FITC and TRITC filters causing the merge to appear yellow, whereas true GFP expression only appears in the FITC filter and is green.

Previous studies showed that the only functional NOX/DUOX in *C*. *elegans*, BLI-3, is present in the pharynx, the hypodermis and the intestine of the worms [10, 12]. We postulated that heme peroxidases that might functionally interact with BLI-3 are likely to be in one or more of the tissues that harbor BLI-3. Indeed, we previously demonstrated that SKPO-1, another immune-protective peroxidase, localizes to the hypodermis [16]. To functionally localize HPX-2, we conducted tissue-specific RNAi to knock down *hpx-2* in the intestine or hypodermis and measured the worms’ susceptibility to *E*. *faecalis*. We observed that intestinal RNAi did not affect pathogen susceptibility, whereas hypodermal RNAi resulted in a slight but significant decrease, suggesting that HPX-2 might be present in the hypodermis and contribute to pathogen resistance in this tissue (Fig 3A and 3B).

To directly visualize the expression of HPX-2, we created a partial translational fusion of HPX-2 to GFP. Specifically, the upstream sequence and the first two exons of the *hpx-2* gene were cloned to generate a fusion with GFP, and the construct was injected into the worms (Fig 3C). The resulting extrachromosomal array was then integrated into the chromosome. We initially looked at the stable, transgenic worms at different development stages, but did not observe GFP expression in the majority. Occasionally (in about ~1% of the worms), we observed a green fluorescent stripe extending from the distal bulb of the pharynx to the anterior of the buccal cavity (Fig 3D and 3E). We postulated that the expression level might be too low to detect. In fact, expression data from Wormbase and our RNA-seq data (see below) indicate that the overall expression level of *hpx-2* gene is extremely low at the whole worm level but increases significantly upon entry into dauer. When we subsequently analyzed dauer animals using confocal microscopy, we observed weak expression of *hpx-2*::GFP in the distal bulb of the pharynx in nearly all animals (Fig 3F). At higher magnification, the localization pattern was most consistent with expression in the gland cells (Fig 3G and 3H) [17]. Again, around 1% of the worms showed the striped expression pattern, resembling the structure called the “process” that extends from the dorsal g1 gland cell to the anterior of the buccal cavity (Fig 3E). Interestingly, the process functions in transporting excreted material from the gland cell to the pharyngeal lumen [18, 19]. Overall, these results indicate that *hpx-2* is expressed in the hypodermis and pharynx.

### HPX-2 contributes to cuticle integrity

A functional role in cuticle biogenesis was demonstrated for some of the previously studied heme peroxidases and loss-of-function mutations sometimes caused abnormal cuticle morphology. For example, MLT-7, was shown to function in conjunction with BLI-3 to cross-link the cuticular collagens during the molting process. Loss of MLT-7 resulted in a blistered phenotype due to incomplete cross-linking of these extracellular matrix proteins [13]. Another immune protective peroxidase previously studied by our lab, SKPO-1, has an incomplete penetrance of dumpy phenotype when mutated, again suggesting a role in cuticle generation [14].

**Fig 4:**
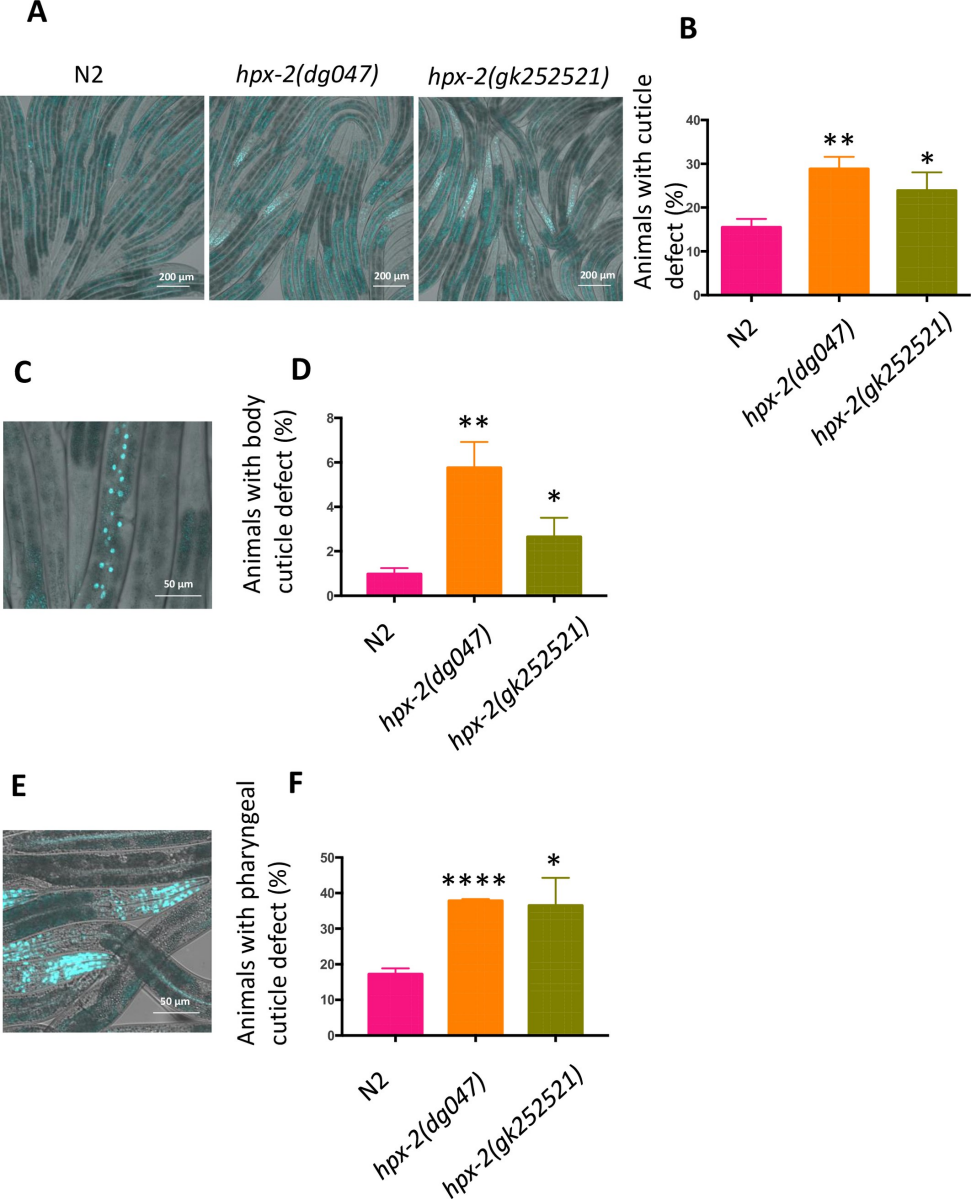
The cuticle defect of *hpx-2* mutants. (A) Hoechst staining of N2, *hpx-2(dg047)* and *hpx-2(gk252521)* worms. Worms with stained nuclei were scored as positive. Images presented are representative of ~300 worms observed. (B) Quantification of Hoechst positive worms in N2, *hpx-2(dg047)* and *hpx-2(gk252521)* worms. Compared with N2, both mutants had significantly higher level of Hoechst staining (P_*hpx-2(dg047)*_ = 0.0022, P_*hpx-2(gk252521)*_ = 0.033). (C) Hoechst staining at higher magnification of hypodermal cells along body. (D) Quantification of animals with Hoechst staining of body hypodermal nuclei (P_*hpx-2(dg047)*_ = 0.0023, P_*hpx-2(gk252521)*_ = 0.0310). (E) Hoechst staining of nuclei in head region, specifically showing a focal plane cutting through the pharynx. (F) Quantification of animals with Hoechst staining of head nuclei (P_*hpx-2(dg047)*_<0.0001, P_*hpx-2(gk252521)*_ = 0.0137). The graphs in B, D and F were generated from 3 independent experiments (n = 100). Error bars represent SEM.

Given the pathogen sensitivity phenotype that resulted from hypodermal-specific RNAi (Fig 3B), we postulated that HPX-2 might play a role in the generation and/or structural integrity of the cuticle. However, unlike the *mlt-7* or *skpo-1* mutants, the *hpx-2* animals displayed normal morphology as observed under the dissecting microscope (S3A Fig). To test for more subtle defects, cuticle integrity was measured by testing how permeable the animals were to a DNA dye [20]. Specifically, we exposed the worms to the DNA staining agent Hoechst 33258 and scored how many exhibited nuclear staining of underlying tissue (Fig 4A). While about 15% of N2 animals showed evidence of nuclear staining, twice as many of the HPX-2 mutants displayed this phenotype, indicative of some loss of cuticle integrity (Fig 4B). The staining was observed at higher resolution to better classify which tissues were affected. Along the length of the worm, we sometimes observed enhanced staining of the hypodermal cell nuclei that underlie the cuticle on the outside of the worm’s body (Fig 4C). We counted animals that were specifically positive for this staining pattern and found that less than 1% of N2 animals were positive for dye penetrance along the body cuticle, but 2–5% of the *hpx-2* mutants were, depending on the allele (Fig 4D). Staining of the hypodermal cell nuclei also occurs in the head region, but at higher resolution additional staining of the pharyngeal muscle tissue was observed, indicative of penetration of the cuticle that lines the pharyngeal lumen (Fig 4E). Approximately twice as much staining was apparent in the *hpx-2* mutants compared to N2 (Fig 4F). These data are consistent with a weaker, more penetrant cuticle in both the hypodermal and pharyngeal cuticle.

Even though the loss of HPX-2 did not affect the low-resolution morphology of the worm, we observed that some of the strains generated by injection of higher amounts of the *hpx-2* complementation construct displayed a slightly dumpy or partial roller phenotype. These animals were also more sensitive to *E*. *faecalis* than wild-type or *hpx-2* mutant animals (S4A and S4B Fig). We hypothesized that overexpression of *hpx-2* might be contributing to these phenotypes. The level of *hpx-2* expression in the different strain backgrounds was measured, showing a correlation between higher mRNA levels and hyper-sensitivity to *E*. *faecalis* (S4C Fig). The results indicate that too much as well as too little HPX-2 cause cuticle defects and sensitivity to the pathogen.

### HPX-2 limits colonization by *E*. *faecalis*, but has no effect on *P*. *aeruginosa*

**Fig 5:**
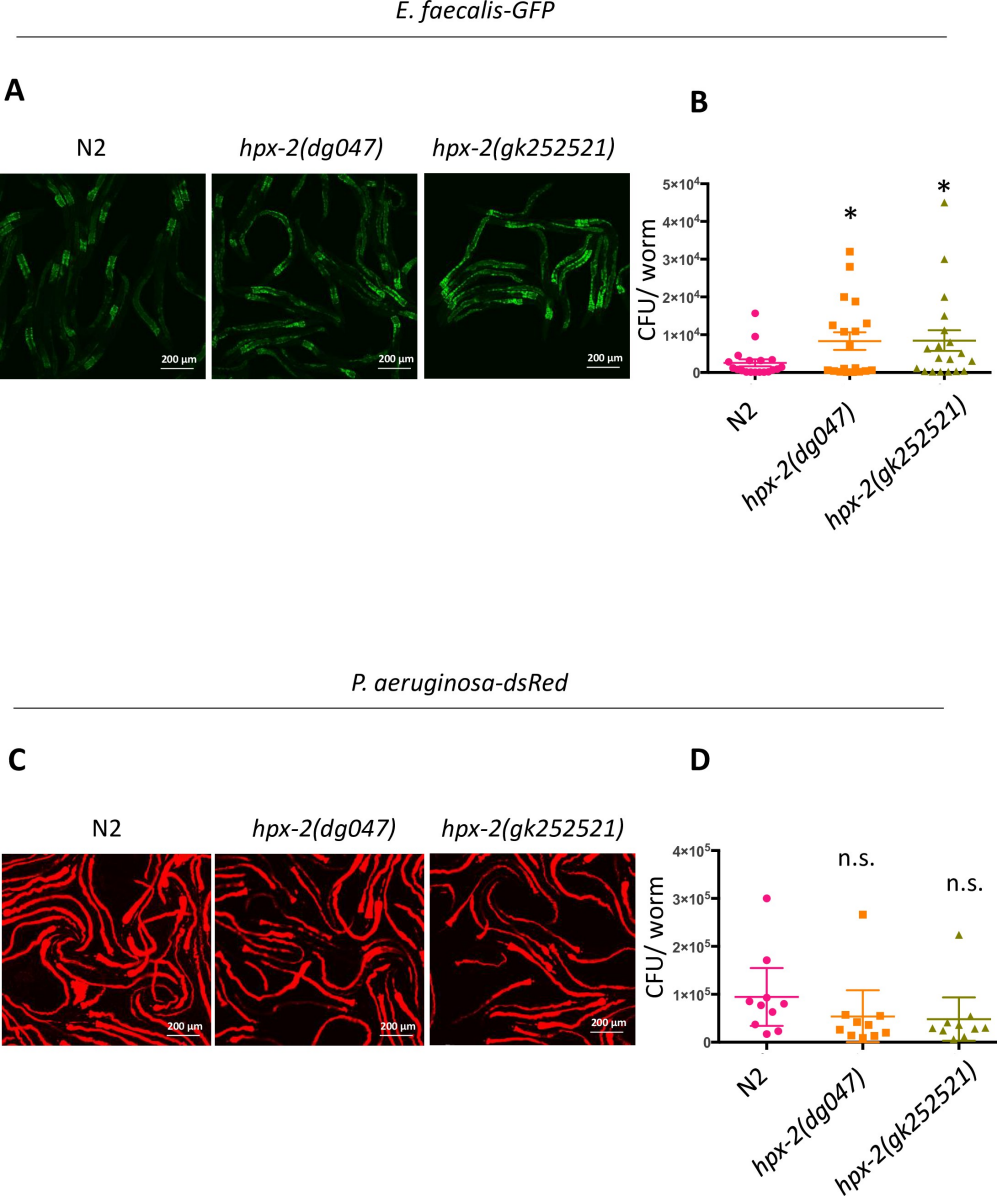
Intestinal colonization of *hpx-2* mutants by pathogens. Colonization of (A) GFP-labeled *E*. *faecalis* OG1RF and (C) dsRed labeled *P*. *aeruginosa* PA14 in N2, *hpx-2(dg047)* and *hpx-2(gk252521)* worms at day two and day one of infection, respectively. (B) *E*. *faecalis* CFU counts per worm (P_*hpx-2(dg047)*_ = 0.0471, P_*hpx-2(gk252521)*_ = 0.0275, n = 19 (combined from 3 independent experiments)). (D) *E*. *faecalis* CFU counts per worm (P_*hpx-2*(dg047)_ = 0.1833, P_*hpx-2*(gk252521)_ = 0.2724, n = 19 (combined from 3 independent experiments)). Error bars represent SEM.

Next, the functional relevance of the pharyngeal presence of HPX-2, was further examined. As mentioned, the apical surface of the pharyngeal lumen is lined with cuticle, which connects to the cuticle of the hypodermis. Specialized projections of the pharyngeal cuticle can form structures, such as the teeth-like formations found in the terminal bulb (grinder) that are thought to contribute to mechanical microbial disruption [18]. HPX-2 was found to be expressed in the gland cells of the pharynx (Fig 3D–3G), and the pharyngeal cuticle was more penetrant to Hoescht dye in the *hpx-2* mutants (Fig 4E and 4F). The data is consistent with HPX-2 aiding cuticle development/remodeling of this organ following excretion into the pharyngeal lumen. We hypothesized that loss of *hpx-2* weakens the pharynx, resulting in less disruption of ingested bacteria and increased intestinal colonization by pathogens. To test this hypothesis, we exposed L4 worms to a strain of *E*. *faecalis* OG1RF that constitutively expresses GFP and measured the level of intestinal colonization by both fluorescence microscopy and CFU plating. At Day 2 of infection, we observed a higher level of intestinal colonization in the *hpx-2* mutant strains, indicated by higher fluorescent intensity and CFU counts (Fig 5A and 5B). In contrast, when we exposed worms to a *P*. *aeruginosa* PA14 strain expressing dsRed [21], we did not observe any significant differences between the N2 strain and the mutants in either fluorescent intensity or CFUs (Fig 5C and 5D), which is consistent with the observation that N2 and *hpx-2* mutants are equally susceptible to *P*. *aeruginosa* (Fig 1G). Although the *hpx-2* mutant might have impaired grinding capability, no obvious structural abnormalities of the pharynx were observed when examined by light and high-resolution transmission electron microscopy (TEM) (S5 Fig). TEM also show that the teeth-like structures found in the grinder are normally shaped (S5B Fig). In addition, the pumping rate and brood size of the worms were not significantly affected (S3B and S3C Fig). Altogether, these results suggest that HPX-2 functions in the pharynx to reduce intestinal colonization by some pathogens, possibility through structural reinforcement, hardening, of the pharyngeal cuticle, and not by changing morphological features such as the overall shape of the grinder and the grinder teeth.

### The catalytic activity of HPX-2 is partially required for the resistance to pathogens

Sequence comparison of HPX-2 to other peroxidases indicates that HPX-2 possesses all the conserved residues required for peroxidase catalytic activity, including the proximal histidine (H^240^), the catalytic Arginine (R^372^), and the distal histidine (H^476^) (reviewed by [22]). On the other hand, similar to BLI-3 and MLT-7, HPX-2 lacks the covalent heme-binding residues that are conserved in mammalian peroxidases [22]. However, this does not rule out the possibility of non-covalent heme binding since both human DUOX1 and 2 lack the conserved residues but still bind heme weakly [23, 24]. Thus, we hypothesize HPX-2 has peroxidase activity.

In previous work, a *skpo-1* mutant was characterized as releasing more H_2_O_2_ than N2 when exposed to the pathogen *E*. *faecalis*, presumably because less H_2_O_2_ was being utilized [14]. In contrast, we found H_2_O_2_ levels generated by the *hpx-2 (dg047)* mutant were comparable to N2 following exposure to *E*. *faecalis* (S6 Fig). While this could be because HPX-2 possesses little peroxidase activity, it also could be due to its low expression level or a lack of activity under these conditions.

**Fig 6:**
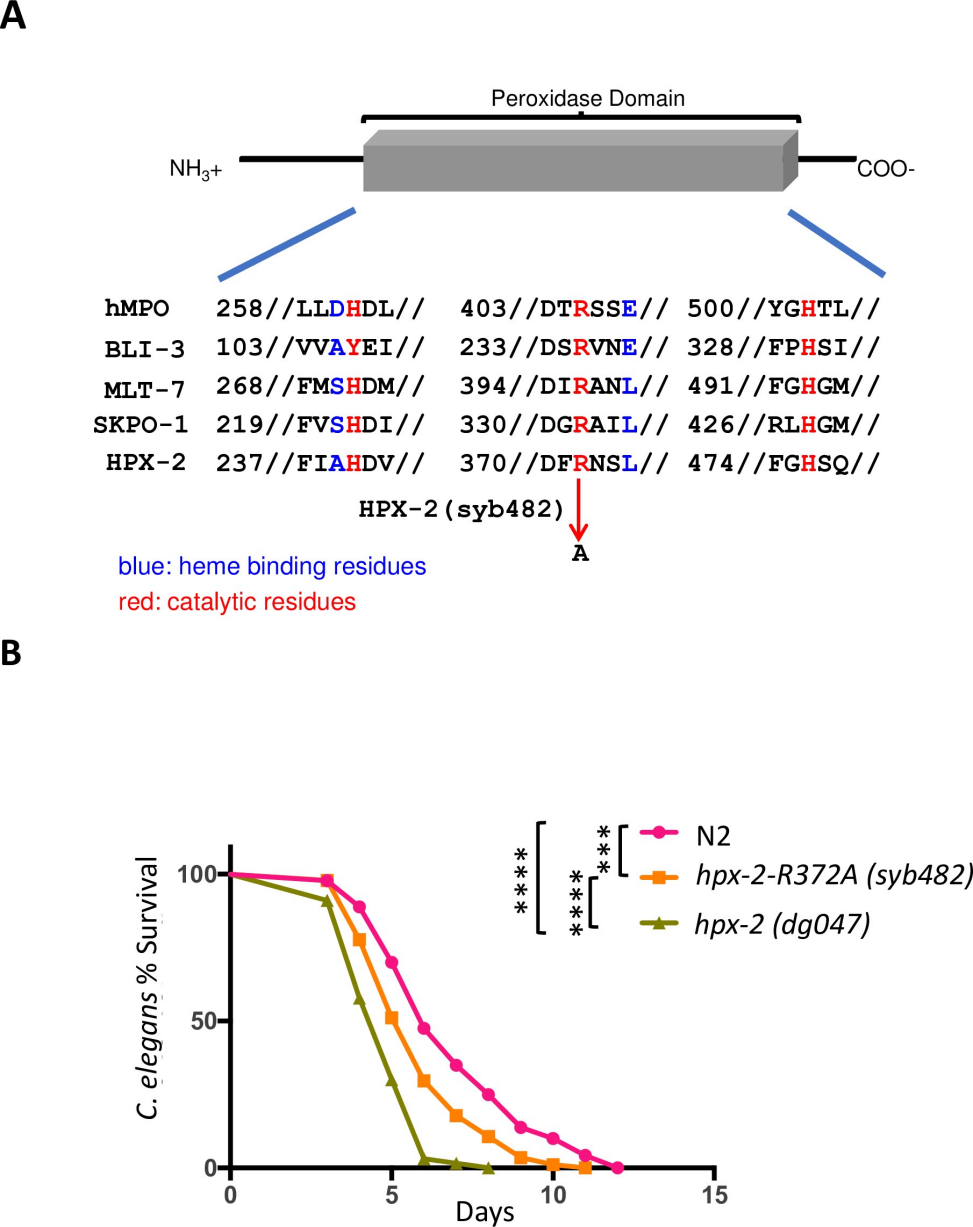
HPX-2 peroxidase activity is required for pathogen resistance. (A) Sequence alignment of the peroxidase domain of HPX-2 and HPX-2 site-directed (SD) mutant to other peroxidases and DUOX. hMPO, human myeloperoxidase. (B) The survival of N2, HPX-2(R372A) strain *(hpx-2(syb238)*), and *hpx-2* mutant strain (*hpx-2(dg047))* on *E*. *faecalis*. Results from one representative experiment with an n of approximately 90 worms for each condition are shown. Median survival and P-values are listed in S8 Table as experiment No.1 along with those from additional biological replicates.

To further examine the possible contribution of HPX-2’s peroxidase activity to pathogen resistance, we analyzed a strain harboring a single amino acid mutation that changes an active site residue of the peroxidase domain. Specifically, the catalytic arginine residue was substituted to alanine using CRISPR-Cas9 mediated mutagenesis (S7 Fig). The catalytic arginine is conserved throughout the peroxidase–cyclooxygenase superfamily, and the substitution of the arginine to alanine abolishes peroxidase activity [25, 26]. The point mutant exhibited increased sensitivity to *E*. *faecalis*, however, the phenotype was not as strong as the deletion mutant (Fig 6B). These data suggest that the catalytic activity of the peroxidase active site of HPX-2 contributes to the pathogen resistance phenotype.

### Transcriptome analysis reveals that *hpx-2* mutant misregulates genes for cuticle synthesis and has a stronger response to infection

**Fig 7:**
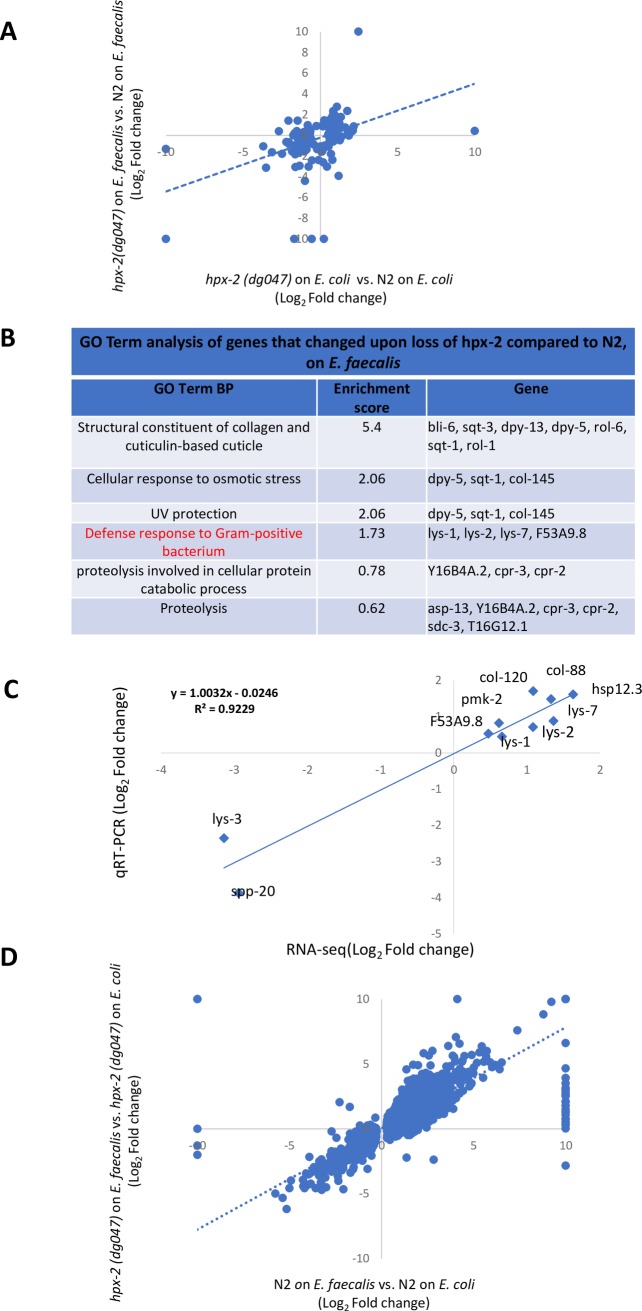
Transcriptome analysis of *hpx-2* mutant. (A) Scatter plot of genes changed in *hpx-2(dg047)* animals, as compared to N2 animals, when either exposed *to E*. *coli* (x-axis) or *E*. *faecalis* (y-axis). Values of 10 or -10 indicate expression level under one condition was 0 RPKM. (B) Gene Ontology (GO) term of Biological Process (BP) analysis of genes that changed upon loss of *hpx-2(dg047)* compared to N2, when exposed to *E*. *faecalis*. (C) qRT-PCR validation of 10 selected genes differentially expressed in the RNA-seq analysis of *hpx-2(dg047)* to N2 on *E*. *faecalis*. Mean log_2_ (fold change) determined in three independent qRT-PCR were plotted against the log_2_ (fold change) from the RNAseq experiments. (D) Scatter plot of genes changed when exposed to *E*. *faecalis*, as compared to *E*. *coli*, in N2 (x-axis) or *hpx-2(dg047)* (y-axis) animals. Genes that are significantly changed under at least one condition are presented. Values of 10 or -10 indicate expression level under one condition was 0 RPKM.

To better understand how the loss of HPX-2 affected the global gene profile of the worm, we conducted a transcriptome analysis using RNA sequencing. Specifically, we first examined the differences in gene expression of a *hpx-2* mutant (*hpx-2 (dg047))* compared to the N2 reference strain under both non-pathogenic (exposed to *E*. *coli* OP50) and pathogenic (exposed to *E*. *faecalis* OG1RF) conditions. Genes that were significantly altered in *hpx-2* animals are plotted in Fig 7A. Under the non-pathogenic condition (exposed to *E*. *coli* OP50), there were a total of 69 genes that were significantly differentially expressed in the mutant, with 34 genes up-regulated and 35 down-regulated (S1 Table). 21 of the affected genes encode for structural constituents of cuticle, including 17 *col* genes, 3 *dpy* genes (*dpy-3*, *dpy-4*, and *dpy-5*), and 2 *rol* genes (*rol-1* and *rol-6*). All of them are upregulated, suggesting a perturbed cuticle biogeneration process upon the loss of HPX-2 (S1 Table). We next analyzed gene expression in a *hpx-2* mutant after 16 hours of exposure to *E*. *faecalis* OG1RF. We chose this early time point because the worms show limited damage and thus the indirect effects should be smaller than later during infection. Under these conditions, there were a total of 125 genes that were differentially expressed in the mutant (72 up-regulated and 53 down-regulated) with 106 of them unique to the pathogenic condition (S2 Table). Interestingly, genes involved in cuticle generation were again significantly altered. Compared to the 21 genes under the nonpathogenic conditions, a total of 41 cuticle genes were affected and up-regulated. In addition to the increased changes in cuticle structural genes, there was an enrichment of genes encoding proteins involved in defense response to Gram-positive bacteria (Fig 7B). These include 4 out of 10 lysozyme genes (*lys-1*, *lys-2*, *lys-3*, and *lys-7)*, *and f53a9*.*8*, which is involved in defense response against Gram-positive bacteria and expressed in the intestine [27–29].

Although the transcriptome changes we detected were modest, both in the number of genes affected and the amplitude of the effect, several observations suggest these reflect the biological effect of HPX-2. First, *hpx-2* gene expression is restricted to a few cells, and thus we would not expect large transcriptome effects that are averaged over the whole animal. Second, we performed five biological replicates of RNAseq and the differences were highly reproducible. Third, the affected genes are enriched for cuticle biogenesis, which correlates well with the known requirement for peroxidases in cuticle cross-linking and the increased permeability of the cuticle to dye that was observed (Fig 4). Fourth, although the overlap in genes that were significantly affected in the *hpx-2* mutant under the two conditions is modest, in most cases genes that were significantly affected in one direction under the *E*. *coli* condition were affected in the same direction under the *E*. *faecalis* condition and vice versa, even if the effect reached significance only in one comparison (Fig 7C). Finally, we used qRT-PCR to validate ten genes that were changed in the *hpx-2* mutant compared to N2 under pathogenic conditions. These qRT-PCR experiments were performed on RNA isolated from three biological replicates that were independent of the RNAseq RNA samples, yet there was a strong correlation between the results (Fig 7C; R^2^ = 0.92 between fold change by RNAseq and qRT-PCR). Therefore, we conclude that there are modest but significant effects on the transcriptome that implicate HPX-2 in cuticle biogenesis and defense against Gram-positive bacteria.

Next, we examined the gene profiles of animals exposed to *E*. *faecalis* as compared to those exposed to OP50 (Fig 7D, S3 and S4 Tables). In N2, a total of 4217 genes (2560 up-regulated and 1657 down-regulated) were significantly differentially expressed, many of which were consistent with previously published data (S3 Table) [30]. In the *hpx-2* mutant, however, a larger number of genes were significantly differentially expressed: 8313 genes in total with 4255 of them up-regulated and 4058 down-regulated (S4 Table). To examine the relationship between the two sets of genes, genes that were significantly expressed under at least one condition were plotted (Fig 7D). Compared to the N2 strain, the *hpx-2* mutant had similar, but stronger responses to the pathogen, contributing to the larger number of genes with significant changes in expression under the pathogenic condition. In summary, we conclude that the loss of *hpx-2* results in a similar, but stronger response to infection. We speculate that the greater microbial load of *E*. *faecalis* observed in the *hpx-2* mutant (Fig 5A) caused this observation of a more robust transcriptional response. In other words, the *hpx-2* animals are essentially farther along in the infectious process than the N2 animals when they were assayed because they controlled the microbial load more poorly.

## Discussion

**Fig 8:**
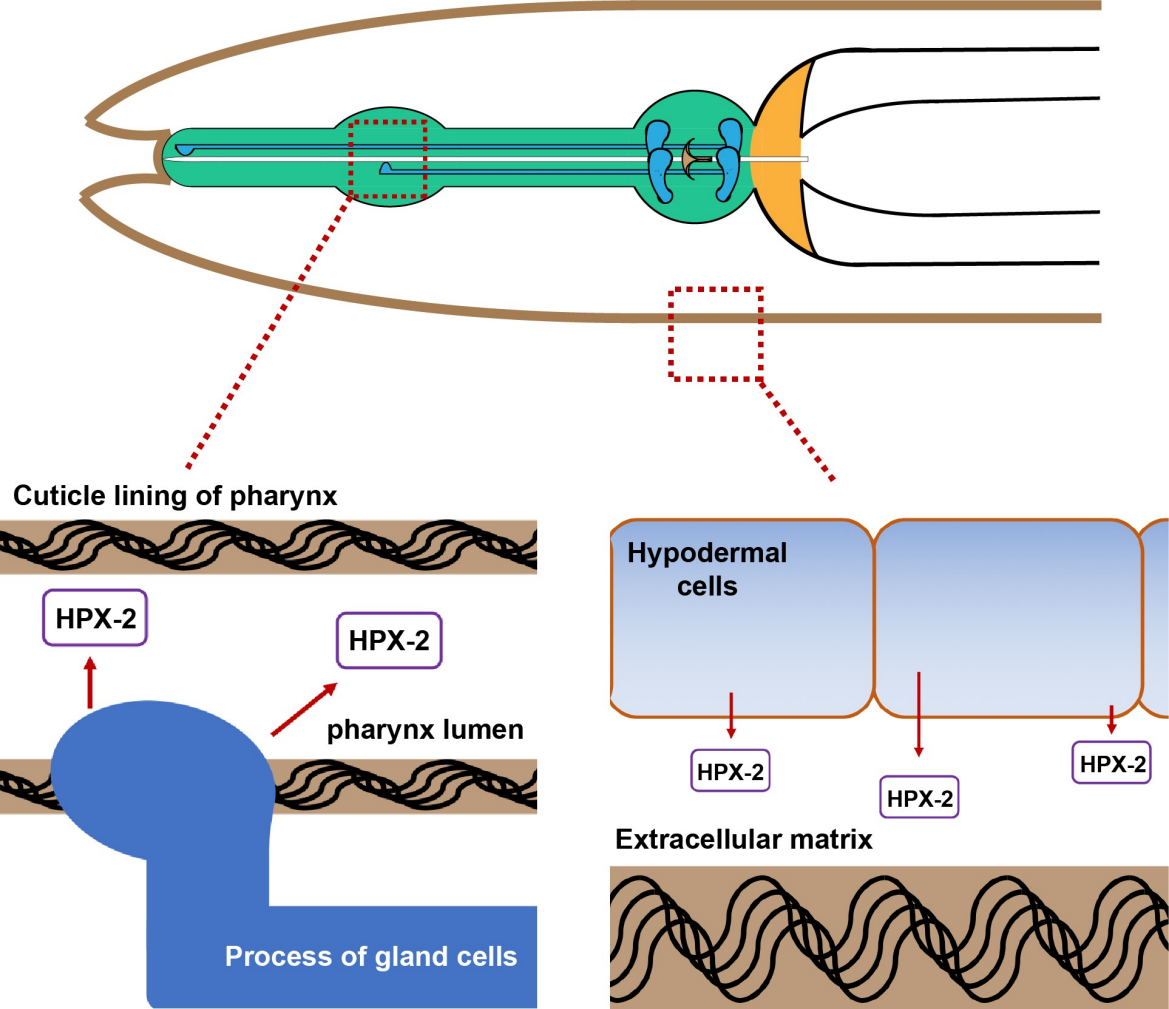
Model for HPX-2 function in hypodermis and pharynx. Diagram of head of the worm. The green structure is the pharynx. Brown lines on the outside of the worm as well as in the pharyngeal lumen indicate the cuticle material that makes up the hypodermal cuticle and pharyngeal cuticle, respectively. The blue globular structures in the terminal bulb of the pharynx are the gland cells, and the blue line extending from them represent the processes through which vesicles travel for export of material to the lumen. We postulate that HPX-2 is released from the gland cells for building of the pharyngeal cuticle and released from the underlying hypodermal cells for building of the hypodermal cuticle. See main text for details.

In this study, we demonstrate that the heme-containing peroxidase HPX-2 plays an important role in attenuating infection by a variety of pathogens. We postulate that HPX-2 functions in two tissues, the pharynx and the hypodermis, to protect worms from infection as modeled in Fig 8. In the pharynx, we favor a model in which HPX-2 contributes to the hardness/impermeability of the grinder, allowing better disruption of microbes with thick cells walls, and inhibiting microbial colonization of the intestine. In the hypodermis, we postulate that HPX-2 also contributes to the structural integrity of the cuticle, and changes to this barrier tissue perturb signaling pathways resulting in greater susceptibility. We discuss both these aspects of HPX-2 function in more detail below.

### Pharyngeal function of HPX-2

In the pharynx, the expression pattern of *hpx-2*::*GFP* resembles that of the gland cell reporter gene B02807::GFP [27, 31], with expression being observed in the process that extends from the terminal bulb to the posterior end of the buccal cavity. There are two groups of gland cells, three g1 cells and two g2 cells, located at the distal bulb of the pharynx. The gland cells are thought to secrete vesicles containing enzymes that function in the digestion of microbial food and aid in cuticle formation during the molting process [19]. Specifically, during the feeding process, small vesicles are transported from the g1 cells through the process to the secretory ducts to aid digestion. During molting, much larger vesicles are transported through the process and the enzymes they contain are thought to be involved in the tearing down and building up of the cuticle, a critical remodeling process [19].

HPX-2 has a signal peptide sequence and is predicted to be secreted. We postulate that HPX-2 is protective by virtue of it being released into the pharyngeal lumen by gland cells to aid in cuticle remodeling of the pharynx during development. A properly developed pharyngeal cuticle is likely necessary for effective disruption of microbes during the digestive process. Because Gram-positive bacteria and fungi like *C*. *albicans* have thicker, harder-to-disrupt cell walls, this model could explain why the *hpx-2* mutants were colonized more efficiently by, and were more susceptible to, these pathogens compared to Gram-negative bacteria (Figs 1 and 5). The increased microbial load of *E*. *faecalis* in the *hpx-2* mutant could also explain why the transcriptional response was stronger compared to N2 (Figs 5A, 5B and 7D). The cell envelope of Gram-negatives is comprised of two membranes, but only a thin layer of peptidoglycan, rendering these organisms easier to disrupt by mechanical means (Reviewed in [32]), and may explain why the *hpx-2* mutants were not more susceptible to them. While we favor HPX-2 function ultimately affecting the mechanical efficiency of pharyngeal grinding, other explanations such as helping generate a microbicidal oxidant or effects on immune signaling remain formally possible.

In previous studies examining staged animals, significant levels of *hpx-2* expression were only observed during dauer [33]. Using lines expressing an HPX-2::GFP fusion, we also detected expression during dauer in most animals examined (Fig 3E). However, we did not observe GFP expression in the vast majority of animals at other stages (L1-L4 and adult) and during pathogen exposure. The *hpx-2* transcript was also not induced following pathogen exposure (S3 Table). Expression was observed only occasionally in the pharyngeal processes as shown in Fig 3D. Other genes encoding proteins involved in cuticle development like *mlt-7* are expressed at intervals corresponding to the larval molts [13]. However, we did not observe consistent expression associated with molting using staged HPX-2::GFP animals. Based on all these observations, it appears that *hpx-2* is expressed during dauer, a time of cuticle remodeling, but the timing of its role in non-dauer development remains unclear. It is possible that HPX-2 is subject to mechanisms of post-transcriptional regulation that would not necessarily be elucidated by these approaches focused on transcriptional regulation.

### Hypodermal function of HPX-2

Peroxidases play important roles in cuticle bio-generation process and loss-of-function mutants frequently exhibit morphological defects. Mutations in MLT-7 or the peroxidase domain of BLI-3 result in severe blister phenotypes [13, 34]. *skpo-1* mutants have a partially penetrant dumpy phenotype [14]. Loss of HPX-2 did not result in a morphological phenotype observable by light-microscopy or TEM. However, the increase in cuticle permeability, as evidenced by Hoechst staining, indicates some perturbation of the cuticle. Additionally, overexpression of *hpx-2*, by injection of higher concentrations of the transgene, resulted in some *dpy* and *rol* animals, again indicative of HPX-2 playing a role cuticle biogenesis in the hypodermis. Tissue-specific RNAi knock-down of *hpx-2* in the hypodermis resulted in a weak susceptibility phenotype. Collectively, these data suggest that *hpx-2* is expressed and plays an infection-protective role in the hypodermis, though expression of our HPX-2::GFP transgene was not detectable in this tissue.

How might a hypodermal cuticle impaired by lack of HPX-2 translate into susceptibility to pathogen, particularly pathogens that colonize and infect the intestine? First of all, it is important to note that not all cuticle defects alter sensitivity. For example, *mlt-7* mutations and *bli-3* peroxidase domain mutations result in severe blistered phenotypes, with no concomitant increase in pathogen susceptibility [8, 14]. Interestingly, a recent study showed that disruption of some, but not all, aspects of hypodermal cuticle structure triggers the activation of multiple stress response pathways [35]. Specifically, it was discovered that disruption of the annular furrows activates detoxification, osmolyte sensitivity, and most importantly, antimicrobial responses. In regards to HPX-2, we hypothesize that structural disturbance of the hypodermal cuticle perturbs signal transduction related to innate immune and/or stress responses. These changes in signaling in the hypodermis could affect the responses in other tissue by cell non-autonomous signaling events. However, a precise understanding of how cuticular localized HPX-2 impacts innate immunity will require more detailed knowledge of exactly how HPX-2 modulates cuticle structure.

### Summary

In conclusion, we report the characterization of a heme peroxidase, HPX-2, that functions protectively against multiple pathogen infections in *C*. *elegans*. Our data suggest that HPX-2 contributes in a peroxidase-dependent manner by modulating cuticle structure, affecting barrier function and possibly signaling. Such functions are consistent with the role of peroxidases in other systems. For example, plant peroxidases function during infection in barrier tissue remodeling, specifically cell wall cross-linking and cell wall expansion [36]. Additionally, it has been shown in *Drosophila* that ROS contribute to barrier defense when utilized by peroxidases, but also serve as a signal to induce global production of antimicrobial peptides [37, 38]. This work highlights the importance of peroxidases in such roles extends to *C*. *elegans*.

## Materials and methods

### Strain maintenance

*C*. *elegans* strains were grown and maintained as previously described [39]. The *hpx-2* nonsense mutation strain VC20223 *hpx-2* (*gk252521*) was obtained from the *Caenorhabditis* Genetic Center and was backcrossed with N2 Bristol six times. *C*. *elegans* strains, bacterial strains, and fungal strains used in this study are listed in Supplemental Material, S5 Table. For experiments requiring synchronized worms, L1 stage worms on starved plates were washed off, filtered through a 10μm filter (pluriSelect, pluriStrainer 10μm), harvested by centrifugation, transferred to seeded plates, and grown to the desired stage.

### Strain construction

To generate the *hpx-2(dg047)* CRISPR knock-out strain, single guide (sg) sequences were designed by WU CRISPR (http://crispr.wustl.edu) using the un-spliced sequence of the *hpx-2* gene. Four 18 bp sg sequences with WU score higher than 65 were selected and separately cloned into pJW1219 [40]. A mixture of four plasmid constructs was injected into N2 worms at a concentration of 10 ng/μl each, and with 10 ng/μl of pJW1219-*dpy-10* as a co-CRISPR marker. Worms that displayed a roller or dumpy phenotype were then isolated for genotyping to test for insertions and/or deletions (INDEL) in the *hpx-2* gene. Verified *hpx-2* mutants were backcrossed with N2 to eliminate the *dpy-10* mutation.

The HPX-2 catalytic residue mutant strain (HPX-2-R372A), was generated by Suny Biotech (http://www.sunybiotech.com/) using CRISPR-Cas9. The mutant strain PHX782 *hpx-2(syb782)* was verified by PCR sequencing and the primers are listed in S6 Table.

To generate the *hpx-2* complemented strains, a 12 kb DNA fragment containing the *hpx-2* gene and 5 kb of upstream and 4 kb of downstream sequences was amplified from the genomic DNA of N2 worms. The PCR product was then purified and injected into *hpx-2(dg047)* and *hpx-2(gk252521)* at 20 ng/μl concentration with 50 ng/μl of EcoRV-digested genomic DNA from N2 worms. pCFJ90 (*pMyo2*::*mCherry*) was used as a fluorescent co-injection marker at a final concentration of 2 ng/μl.

To visualize *hpx-2* expression, the GF203 strain was generated by injecting the pPD95.75 (50 ng/μl) plasmid containing the 4 kb upstream of *hpx-2* and the first two exons fused to *gfp*, with 20 ng/μl pRF4 (*rol-6(su1006)*) as a roller co-injection marker. The obtained strains with extrachromosomal arrays were then exposed to trimethylpsoralen (TMP) with UV irradiation and back crossed six times to generate stable integrated transgenic lines using a previously described protocol [41]. All the oligonucleotide primers used in this study are listed in S6 Table.

### Killing and longevity assays

Killing assays and longevity assays were conducted as previously described, with some slight modifications [42–44]. All assays were done with 30 worms at the L4 stage on three replica plates (or 6-plate wells for the *C*. *albicans* assay) for a total of 90 animals and scored for survival over time. For plate preparation, *E*. *faecalis* OG1RF was grown in BHI media for 5 hours and seeded onto BHI agar plates with 50 μg/ml of gentamycin and grown overnight at 37°C. For the vancomycin inactivated killing assay, *E*. *faecalis* OG1RF was grown in BHI medium overnight and concentrated 10-folds prior to being seeded onto BHI agar plates with 50 μg/ml of gentamycin and 15 μg/ml of vancomycin and incubated for 5 hours at 37°C. *P*. *aeruginosa* PA14 was grown in LB broth overnight and seeded onto SK plates, incubated at 37°C for 24 hours, and then 25°C for another 24 hours. *S*. *enterica* SL1344 was grown in LB overnight and seeded onto SK plates and incubated at 37°C overnight. *S*. *aureus* NCTC8325, grown in TSB with 10 μg/ml nalidixic acid (Nal) overnight, was seeded to TSA+Nal plates and incubated at 37°C for 6 hours. *C*. *diphtheriae* NCTC13129 grown in BHI overnight was seeded onto BHI plates containing 25 μg/ml Nal and 50 μg/ml 5-fluoro-2-deoxyuridine (FuDR) and incubated at 37°C for 24 hours. *C*. *albicans* strain SC5314 was grown overnight in BHI media and spotted onto solid BHI plates. L4 stage worms were exposed to *C*. *albicans* for 4 hours and were collected, washed, and transferred to six-well plates containing 20% BHI and 80% M9W, and scored for survival daily [45].

### RNA interference

To knock down *hpx-2* gene expression, L1 to L4 stage larvae were exposed to *E*. *coli* HT115 containing the expression plasmid for double-stranded *hpx-2* previously constructed [16]. L4 stage worms were then transferred to *E*. *faecalis* killing plates for the pathogen susceptibility assay.

### Fluorescence microscopy

To visualize HPX-2::GFP expression, worms with the roller phenotype indicative of the co-injection marker were picked and anesthetized with 25 mM tetramisole and mounted on 2% agarose pads. The worms were then visualized and imaged using Olympus FLUOVIEW FV3000 confocal microscopy equipped with Fluoview FV315-SW software. Z-stack images were acquired using a step size of 0.7 μm and processed using Olympus cellSens Dimension Desktop software.

To visualize intestinal colonization by *E*. *faecalis* and *P*. *aeruginosa*, *C*. *elegans* were exposed to these pathogens for 48 hours (*E*. *faecalis*) and 24 hours (*P*. *aeruginosa*) respectively on agar plates. They were then washed off the plates and washed four times with 1 ml of M9W. Following anesthesia with 25 mM tetramisole hydrochloride, the worms were mounted on 2% agarose pads for imaging, using Olympus FLUOVIEW FV3000 confocal microscopy equipped with Fluoview FV315-SW software.

### Colony forming unit assay of intestinal colonization

To measure intestinal CFUs, animals were exposed to bacteria as described above and then washed off plates with M9W. They were then washed three times in 1 ml of M9W, then twice more with 1 ml of M9W containing 25 mM tetramisole hydrochloride. The worms were then treated with 500 μl M9W containing 25 mM tetramisole hydrochloride, 1 mg/ml of ampicillin, and 1 mg/ml kanamycin for 1 hour to kill all surface bacteria. The treated worms were washed twice more with 1 ml of M9W containing 25 mM tetramisole hydrochloride, transferred to an Eppendorf tube containing 100 μl of M9 (one worm per tube), and disrupted using a motorized pestle (Kontes cordless pestle (cat# K749540-0000) and pestles (cat# K749521-1590) for 1 min. The solution was then serially diluted in M9 and spotted on agar plates for CFU counting.

### Hoechst staining

Hoechst staining of the worms was performed as previously described [46] with the following modifications. Specifically, worms were washed off of plates with M9W buffer, and then incubated in M9W containing 10 μg/ml Hoechst 33258 dye (Sigma) at room temperature for 20 minutes with gentle shaking, followed by three more washes with M9W before imaging. Images were acquired using Olympus FLUOVIEW FV3000 confocal microscopy equipped with Fluoview FV315-SW software. Z-stack images were acquired using a step size of 0.7 μm and processed using Olympus cellSens Dimension Desktop software. Hoechst-positive worms were scored based on staining of the cell nuclei, indicative of cuticle penetration.

### Transmission electron microscopy (TEM)

L4 stage worms were collected and fixed in 3% glutaraldehyde overnight. Samples were then prepared and thin-sectioned for transmission electron microscopy as previously described [45]. Image acquisition was done using a JEOL 1400 transmission electron microscope at 60 kV, equipped with a 2K × 2K Gatan charge-coupled-device camera.

### RNA isolation and qRT-PCR analysis

L4 animals were exposed for 16 hours to the condition of interest and total RNA was extracted using Trizol (Invitrogen) according to the manufacturer’s instructions. RNA samples were treated with Turbo DNA free kit (Applied Biosystems) to eliminate DNA contamination. qRT-PCR was performed as previously described [47]. The actin gene was used as an internal control. Primers used in qRT-PCR are listed in S7 Table.

### RNA sequencing

L4 stage worms were exposed to *E*. *faecalis* or *E*. *coli* for 16 hours and total RNA was extracted for 4 biological replicates. Illumina Hiseq 4000 sequencer with 75 nt pair-ended read format was used to conduct the sequencing. The sequencing reads (ranging from 20 million to 37 million per biological replicate) were quality and adaptor trimmed and mapped to the reference genome (version WBcel235 downloaded from http://ensemblgenomes.org) using Tophat [48]. The expression level (RPKM) of annotated genes was measured using Cufflink [49], and differential expression analysis was conducted using Cuffcompare and Cuffdiff [49] using the gene annotation (Caenorhabditis_elegans.WBcel235.37.gff3 downloaded from http://ensemblgenomes.org). Gene Ontology analysis was carried out by using DAVID (the Database for Annotation, Visualization, and Integrated Discovery) 6.8 [50]. Gene enrichment with a Benjamini adjusted P < 0.05 is listed. Genes that were differentially regulated under different conditions are listed in S1–S4 Tables. The sequencing data are available from the GEO database under accession number GSE124372.

### Amplex red assay

To measure H_2_O_2_ concentration of the worms, the Amplex red assay was performed as previously described [16] using the Amplex Red hydrogen peroxide/peroxidase kit (Invitrogen Molecular Probes, Eugene, OR) with the following modifications: L4 worms were exposed to a bacterial strain for 16 hours and transferred to 96 well plates with 30 worms in each well. A total of 80 mM diphenyleneiodinium chloride (DPI) (TCI, Tokyo) was added to some wells and allowed to incubate for 15 minutes prior to addition of Amplex Reagents. After 1 hour of incubation, fluorescence was measured at 540/590 nm excitation and emission, respectively.

### Pharyngeal pumping rate

To measure the pharyngeal pumping rate of the worms, contractions of the posterior pharyngeal bulb were observed and counted over a 10 second interval for 15 adult animals under a 20X magnification stereo microscope.

### Brood size

To measure the brood size of the worms, 9 L4 stage worms were singled on NGM plates, allowed to lay eggs and transferred to a new plate each day until no more eggs were produced. The offspring on the plates were counted to calculate brood size.

### Statistical analysis

Survival, longevity assays, qRT-PCR, intestinal CFU, and Hoechst staining data were analyzed using GraphPad Prism version 7.0 (GraphPad Software, San Diego). Kaplan-Meier log rank analysis was used to compare longevity and survival curves. An unpaired Student’s t-test was used to determine the statistical significance of the intestinal CFU and Hoechst staining data. In all the experiments, P-values < 0.05 were considered to be significant and are noted in the Figs with one asterisk indicating P < 0.05, two indicating P < 0.01, three indicating P < 0.001 and four indicating P < 0.0001.

## Supporting information

S1 Fig HPX-2 contributes *C. elegans* resistance to *E. faecalis*. Survival of worms on *E*. *faecalis* OG1RF following exposure to vector control (VC) RNAi and *hpx-2* RNAi. Representative results from one experiment with an n of approximately 90 worms for each condition are shown. Median survival and P-values along with replicates are listed in S8 Table.
(PPTX)

S2 Fig HPX-2 does not contribute to the overall fitness of the worm on *E. coli*. Survival of N2 and *hpx-2* mutants on (A) *E*. *coli* OP50 and (B) heat-killed *E*. *coli* OP50. Representative results from one experiment with an n of approximately 90 worms for each condition are shown. Median survival and P-values along with replicates are listed in S8 Table.
(PPTX)

S3 Fig *hpx-2* mutants exhibit normal physiological traits. (A) *hpx-2* mutant adults have the same average size and morphology of N2 worms. (B) *hpx-2* mutants have comparable pumping rates compared to N2 worms. P_*hpx-2*(dg047)_ = 0.071, P_*hpx-2*(gk252521)_ = 0.1147, n = 15. (C) *hpx-2* mutants have comparable brood sizes compared to N2 worms. P_*hpx-2*(dg047)_ = 0.2430, n = 9. Error bars represent the SEM, and P-values were calculated via Student’s paired t-test. Data is representative of three independent replicates.
(PPTX)

S4 Fig Overexpression of HPX-2 results in susceptibility to *E. faecalis*. (A) Survival of N2, *hpx-2(dg047)*, and the strain overexpressing *hpx-2* in *hpx-2 (dg047)* background on *E*. *faecalis* OG1RF. (B) Survival of N2, *hpx-2(gk252521*), and the strain overexpressing *hpx-2* in *hpx-2(gk252521*) background on *E*. *faecalis* OG1RF. (C) qRT PCR showing the relative log_2_ (fold change) of *hpx-2* gene expression levels in *hpx-2(dg047)* overexpression (OE) and *hpx-2 (gk252521)* OE strains compared to N2. The average gene expression of biological triplicates is shown, and the error bars represent SEM.
(PPTX)

S5 Fig *hpx-2* mutants exhibit normal pharyngeal structure. (A) *hpx-2* mutants have normal pharyngeal structure compared to N2 worms by light microscopy. Images are representative of >100 N2, *hpx-2(dg047)* and *hpx-2(gk252521)* worms observed. (B) *hpx-2* mutants have normal pharyngeal structure compared to N2 worms by transmission electron microscopy. Shown are example cross-sections corresponding to the regions marked in the pharyngeal diagram. Images are representative of >50 N2 and *hpx-2(dg047)*, worms observed.
(PPTX)

S6 Fig Amplex red assay of N2 and *hpx-2(dg047)* mutant. Worms were grown to L4 stage and exposed to *E*. *coli* OP50 or *E*. *faecalis* OG1RF for 16 hours at 25°C. The amount of H_2_O_2_ were then measured with or without exposure to 80mM diphenyleneiodinium chloride (DPI). Error bars represent the SEM and P-values were calculated via Student’s paired t-test. Data is representative of three independent replicates.
(PPTX)

S7 Fig Sequences of N2 and *hpx-2*(R372A) mutant (*hpx-2(syb782))*. The mutated nucleotide and resulting amino acid are labeled in red.
(PPTX)

S1 Table Genes that are significantly changed in *hpx-2* mutant compared to N2 when exposed to *E. coli* OP50. (XLSX)

S2 Table Genes that are significantly changed in *hpx-2* mutant compared to N2 when exposed to *E. faecalis* OG1RF. (XLSX)

S3 Table Genes that are significantly changed in N2 worms when exposed to *E. faecalis* OG1RF compared to *E. coli* OP50. (XLSX)

S4 Table Genes that are significantly changed in *hpx-2* worms when exposed to *E. faecalis* OG1RF compared to *E. coli* OP50. (XLSX)

S5 Table Strains used in this study. (XLSX)

S6 Table Oligos used in this study. (XLSX)

S7 Table Primers used in qRT-PCR. (XLSX)

S8 Table Median survival and P-values in killing assays. (XLSX)
