# Supplementary figures and images for: Heme peroxidase HPX-2 protects Caenorhabditis elegans from pathogens
Source: PLoS Genet. 2019 Jan 29;15(1):e1007944. doi: 10.1371/journal.pgen.1007944 (PMC6368334; doi:10.1371/journal.pgen.1007944)

## Slide 1
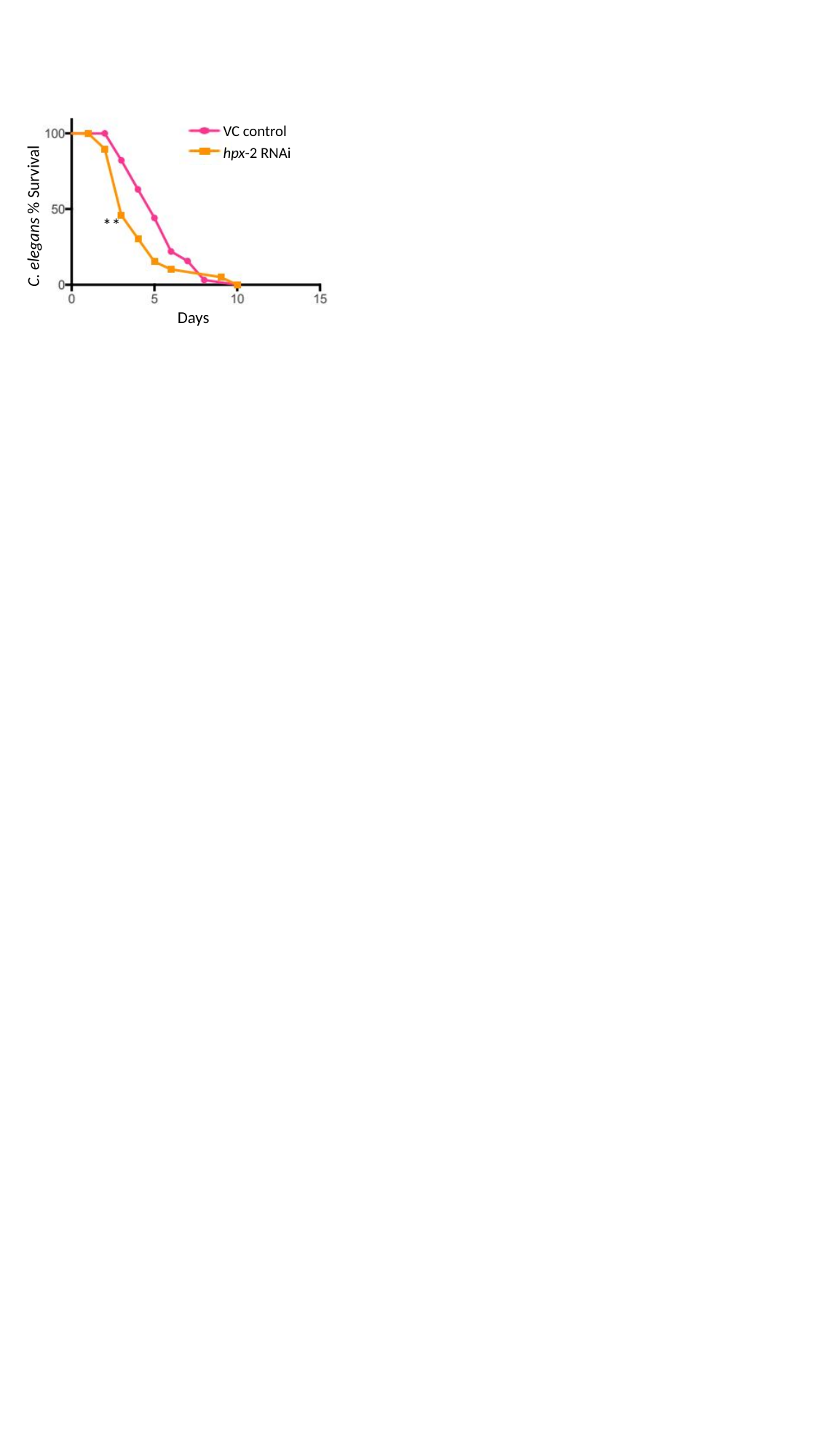

VC control
hpx-2 RNAi
C. elegans % Survival
**
Days

Supplement: S1 Fig — Survival of worms on E. faecalis OG1RF following exposure to vector control (VC) RNAi and hpx-2 RNAi. Representative results from one experiment with an n of approximately 90 worms for each condition are shown. Median survival and P-values along with replicates are listed in S8 Table. (PPTX) [file pgen.1007944.s001.pptx]

## Slide 1
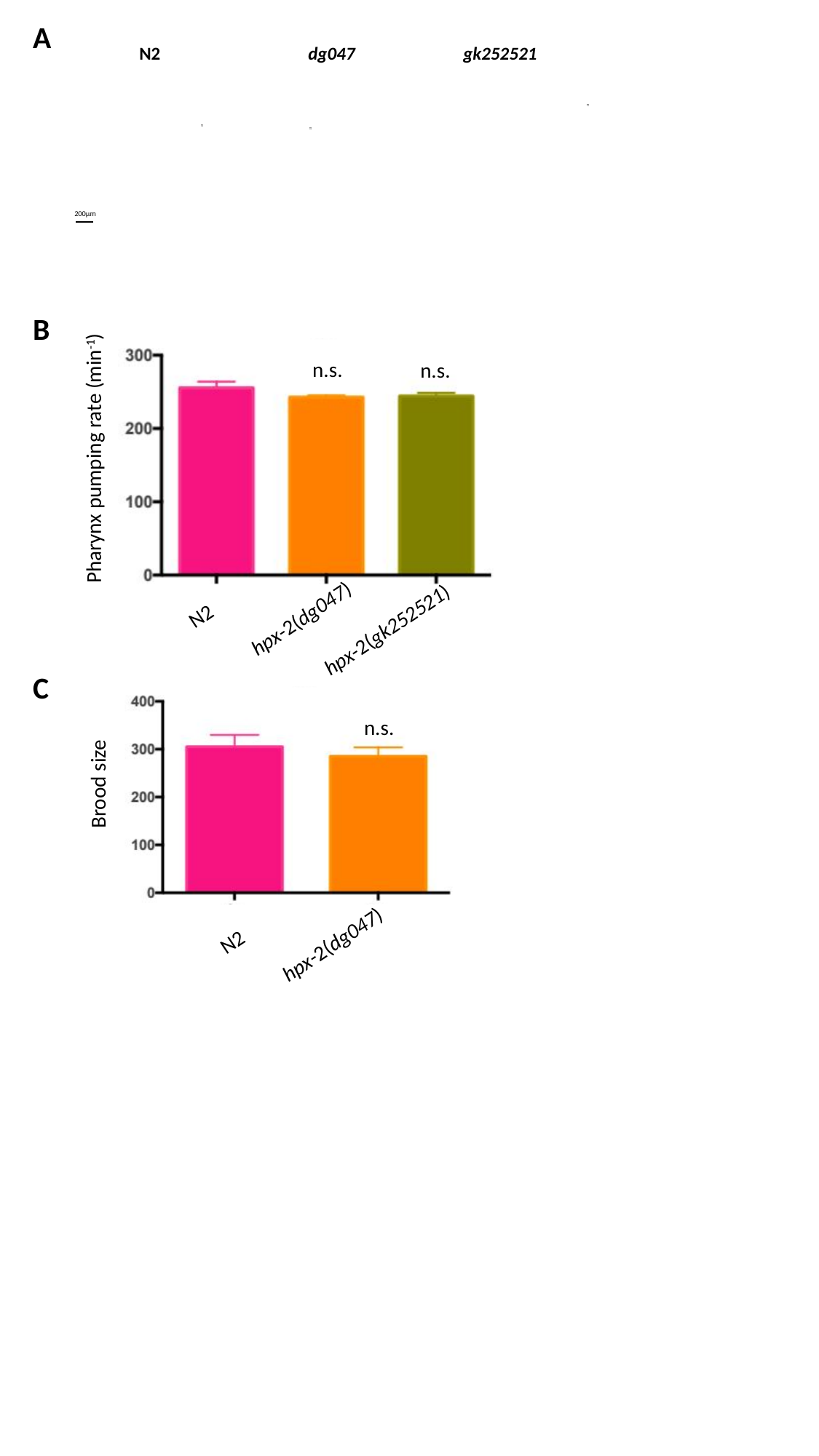

A
dg047
N2
gk252521
200µm
B
n.s.
n.s.
Pharynx pumping rate (min-1)
 N2
hpx-2(gk252521)
hpx-2(dg047)
C
n.s.
Brood size
 N2
hpx-2(dg047)

Supplement: S3 Fig — (A) hpx-2 mutant adults have the same average size and morphology of N2 worms. (B) hpx-2 mutants have comparable pumping rates compared to N2 worms. Phpx-2(dg047) = 0.071, Phpx-2(gk252521) = 0.1147, n = 15. (C) hpx-2 mutants have comparable brood sizes compared to N2 worms. Phpx-2(dg047) = 0.2430, n = 9. Error bars represent the SEM, and P-values were calculated via Student’s paired t-test. Data is representative of three independent replicates. (PPTX) [file pgen.1007944.s003.pptx]

## Slide 1
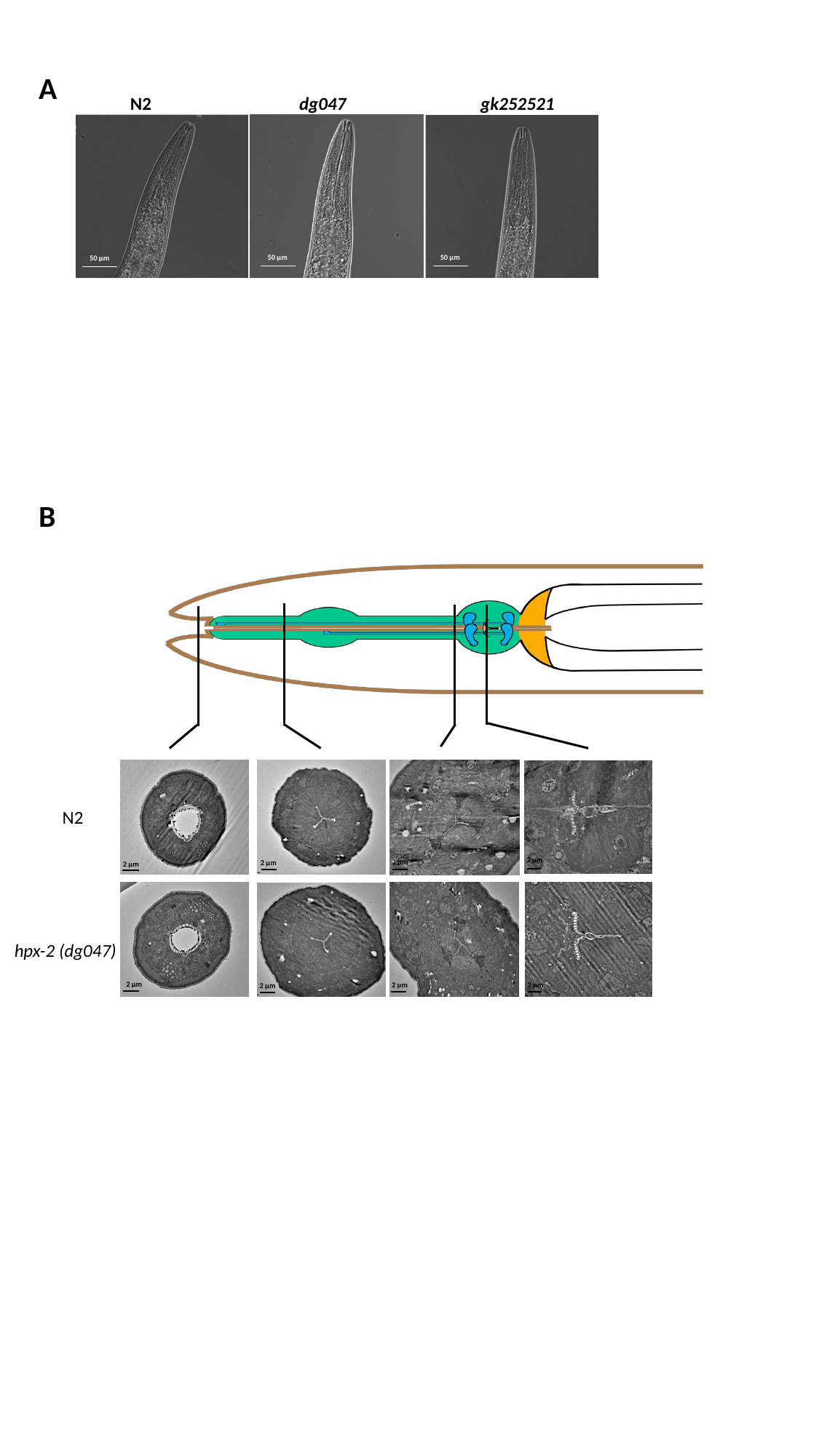

A
N2
dg047
gk252521
50 µm
50 µm
50 µm
B
2 µm
2 µm
2 µm
2 µm
N2
2 µm
2 µm
2 µm
2 µm
hpx-2 (dg047)

Supplement: S5 Fig — (A) hpx-2 mutants have normal pharyngeal structure compared to N2 worms by light microscopy. Images are representative of >100 N2, hpx-2(dg047) and hpx-2(gk252521) worms observed. (B) hpx-2 mutants have normal pharyngeal structure compared to N2 worms by transmission electron microscopy. Shown are example cross-sections corresponding to the regions marked in the pharyngeal diagram. Images are representative of >50 N2 and hpx-2(dg047), worms observed. (PPTX) [file pgen.1007944.s005.pptx]
