# Supplementary material for: Heme peroxidase HPX-2 protects Caenorhabditis elegans from pathogens
Source: PLoS Genet. 2019 Jan 29;15(1):e1007944. doi: 10.1371/journal.pgen.1007944 (PMC6368334; doi:10.1371/journal.pgen.1007944)
Supplement: S2 Fig — Survival of N2 and hpx-2 mutants on (A) E. coli OP50 and (B) heat-killed E. coli OP50. Representative results from one experiment with an n of approximately 90 worms for each condition are shown. Median survival and P-values along with replicates are listed in S8 Table. (PPTX) [file pgen.1007944.s002.pptx]

## Slide 1
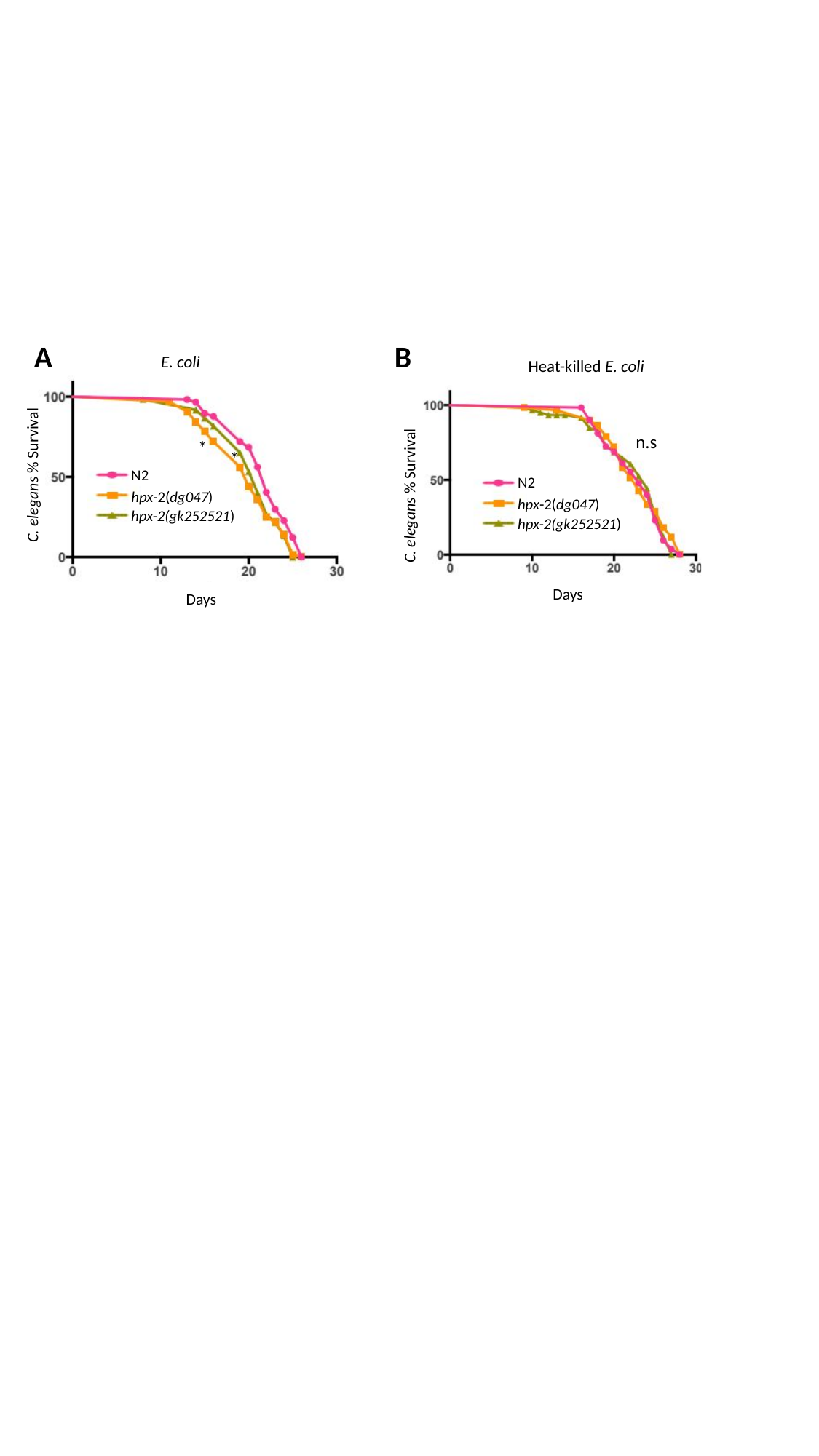

A
B
E. coli
Heat-killed E. coli
n.s
*
*
C. elegans % Survival
N2
hpx-2(dg047)
hpx-2(gk252521)
C. elegans % Survival
N2
hpx-2(dg047)
hpx-2(gk252521)
Days
Days
