# Supplementary material for: Heme peroxidase HPX-2 protects Caenorhabditis elegans from pathogens
Source: PLoS Genet. 2019 Jan 29;15(1):e1007944. doi: 10.1371/journal.pgen.1007944 (PMC6368334; doi:10.1371/journal.pgen.1007944)
Supplement: S4 Fig — (A) Survival of N2, hpx-2(dg047), and the strain overexpressing hpx-2 in hpx-2 (dg047) background on E. faecalis OG1RF. (B) Survival of N2, hpx-2(gk252521), and the strain overexpressing hpx-2 in hpx-2(gk252521) background on E. faecalis OG1RF. (C) qRT PCR showing the relative log2 (fold change) of hpx-2 gene expression levels in hpx-2(dg047) overexpression (OE) and hpx-2 (gk252521) OE strains compared to N2. The average gene expression of biological triplicates is shown, and the error bars represent SEM. (PPTX) [file pgen.1007944.s004.pptx]

## Slide 1
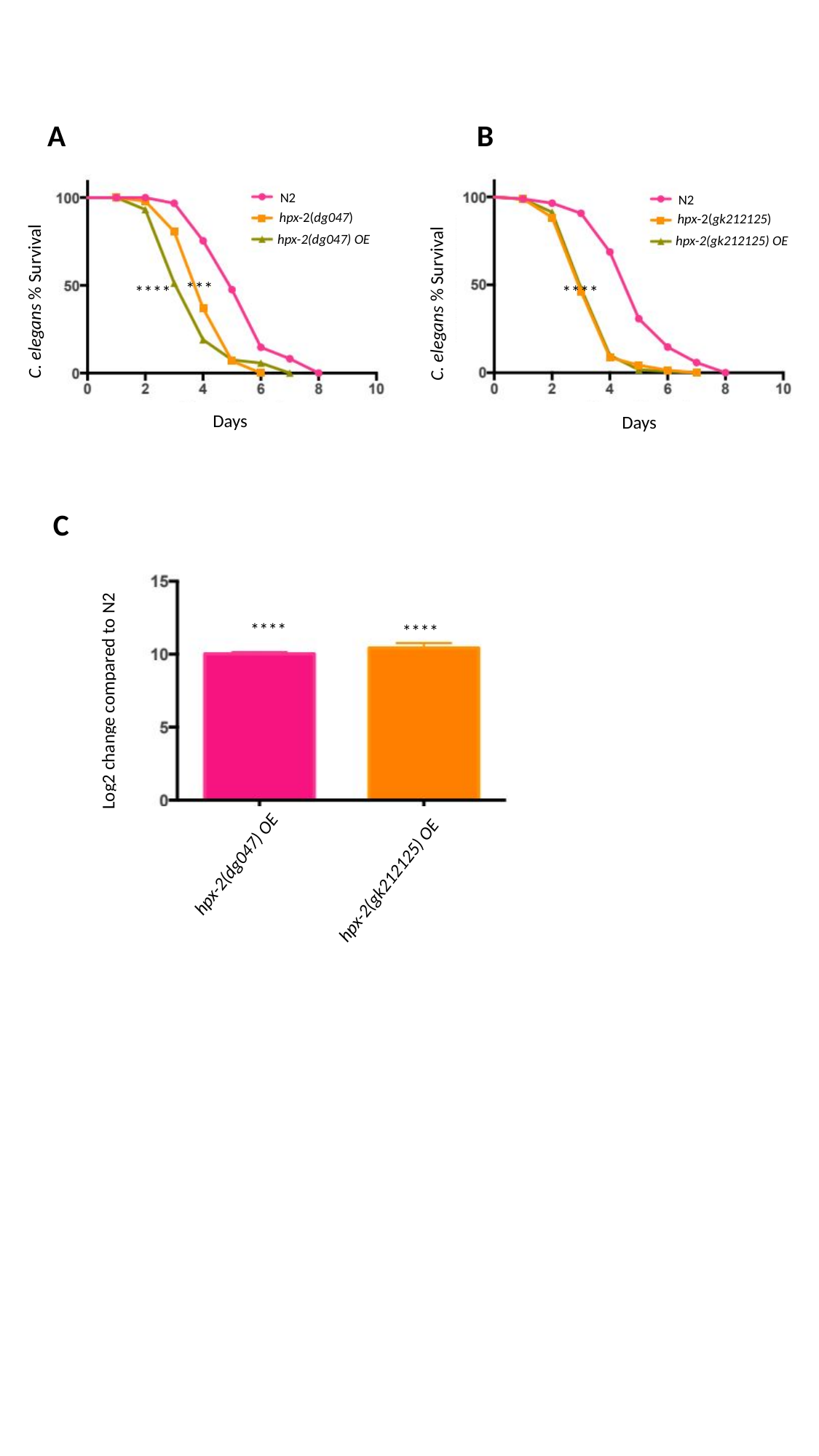

A
B
N2
hpx-2(dg047)
hpx-2(dg047) OE
N2
hpx-2(gk212125)
hpx-2(gk212125) OE
C. elegans % Survival
C. elegans % Survival
***
****
****
Days
Days
C
****
****
Log2 change compared to N2
hpx-2(dg047) OE
hpx-2(gk212125) OE
