# Supplementary material for: Heme peroxidase HPX-2 protects Caenorhabditis elegans from pathogens
Source: PLoS Genet. 2019 Jan 29;15(1):e1007944. doi: 10.1371/journal.pgen.1007944 (PMC6368334; doi:10.1371/journal.pgen.1007944)
Supplement: S6 Fig — Worms were grown to L4 stage and exposed to E. coli OP50 or E. faecalis OG1RF for 16 hours at 25°C. The amount of H2O2 were then measured with or without exposure to 80mM diphenyleneiodinium chloride (DPI). Error bars represent the SEM and P-values were calculated via Student’s paired t-test. Data is representative of three independent replicates. (PPTX) [file pgen.1007944.s006.pptx]

## Slide 1
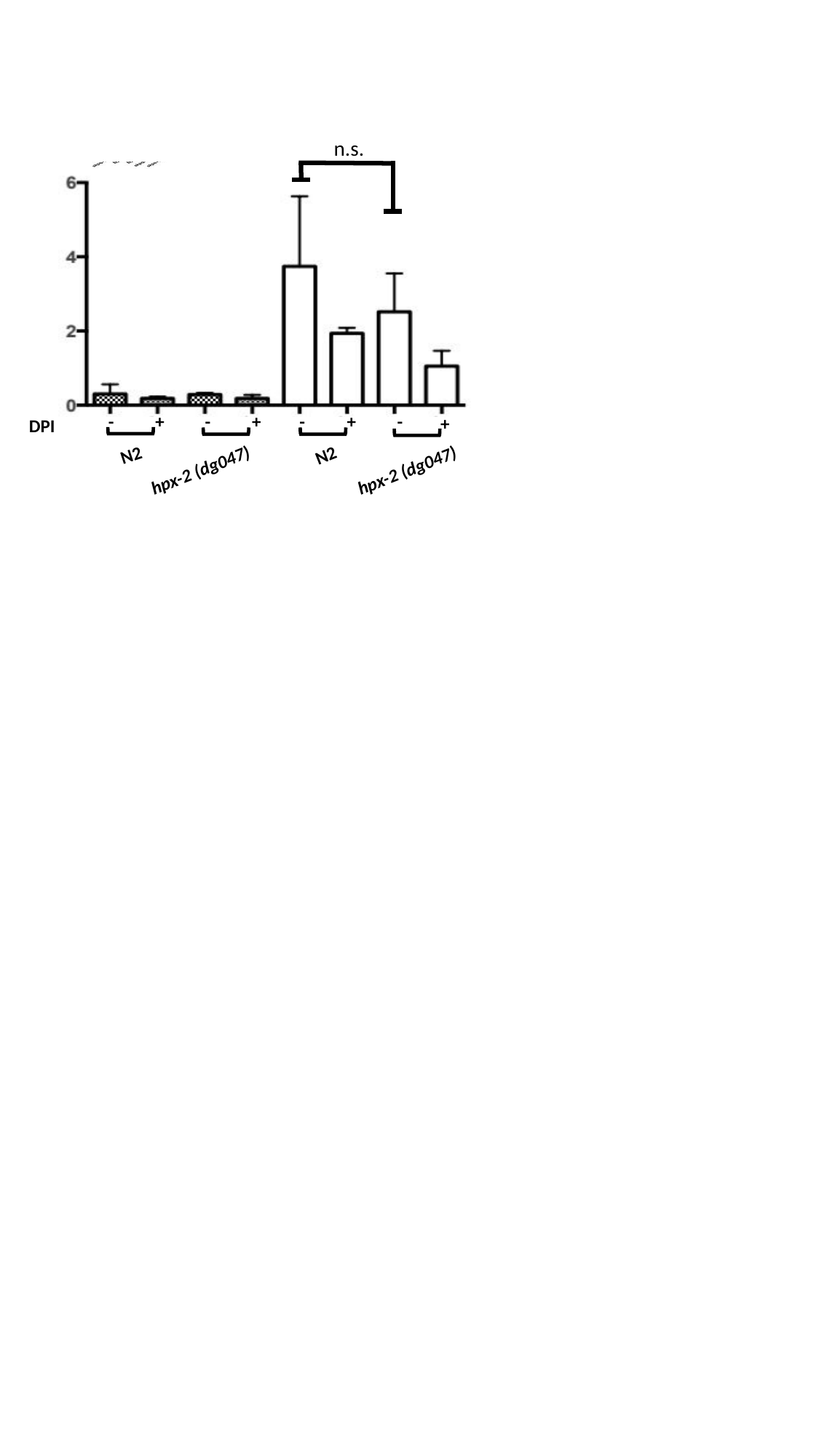

n.s.
-
+
-
+
-
+
-
+
N2
N2
hpx-2 (dg047)
hpx-2 (dg047)
DPI

## Slide 2
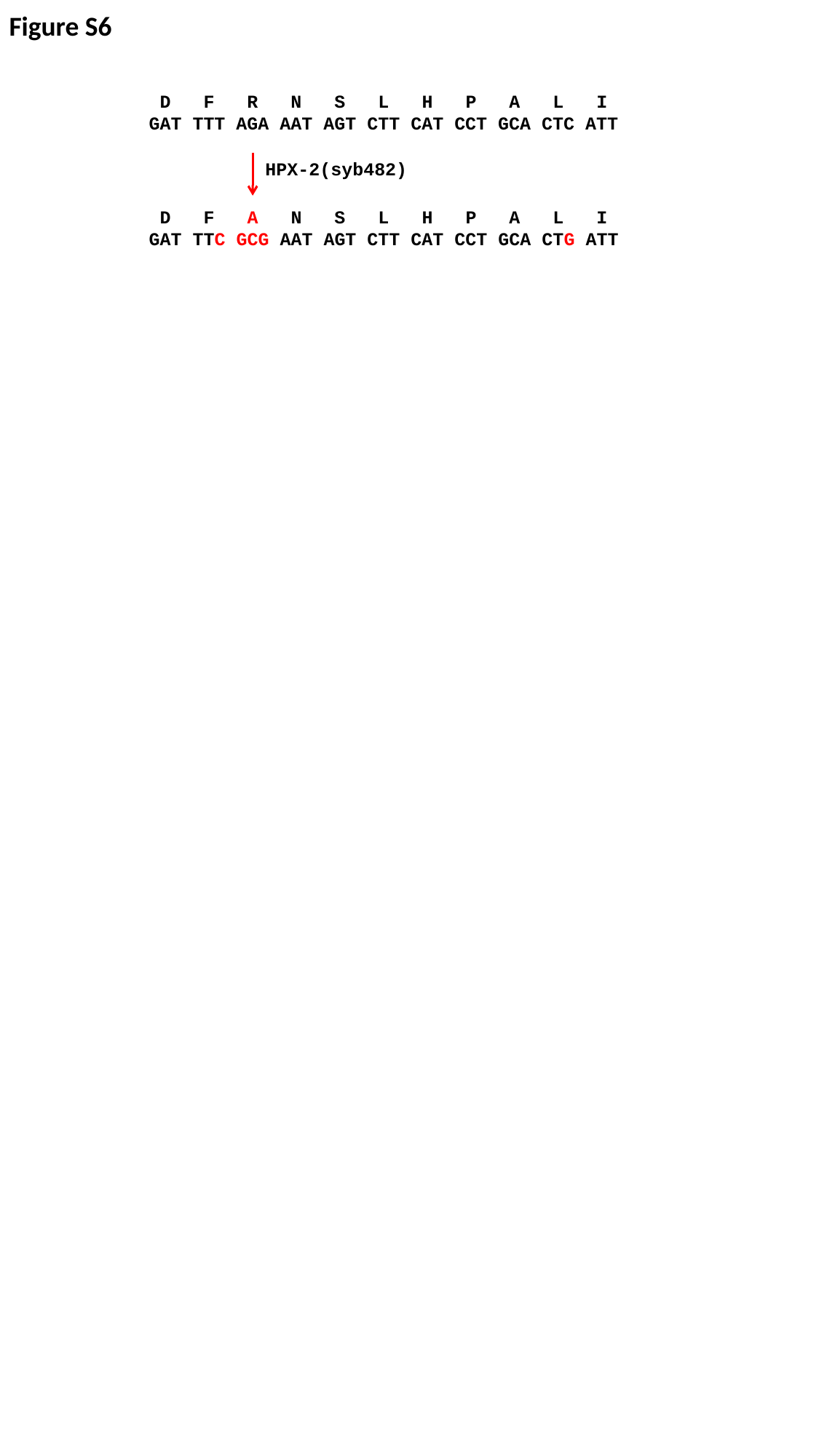

Figure S6
Figure S6
 D F R N S L H P A L I
GAT TTT AGA AAT AGT CTT CAT CCT GCA CTC ATT
HPX-2(syb482)
 D F A N S L H P A L I
GAT TTC GCG AAT AGT CTT CAT CCT GCA CTG ATT
