# Supplementary material for: Heme peroxidase HPX-2 protects Caenorhabditis elegans from pathogens
Source: PLoS Genet. 2019 Jan 29;15(1):e1007944. doi: 10.1371/journal.pgen.1007944 (PMC6368334; doi:10.1371/journal.pgen.1007944)
Supplement: S7 Fig — The mutated nucleotide and resulting amino acid are labeled in red. (PPTX) [file pgen.1007944.s007.pptx]

## Slide 1
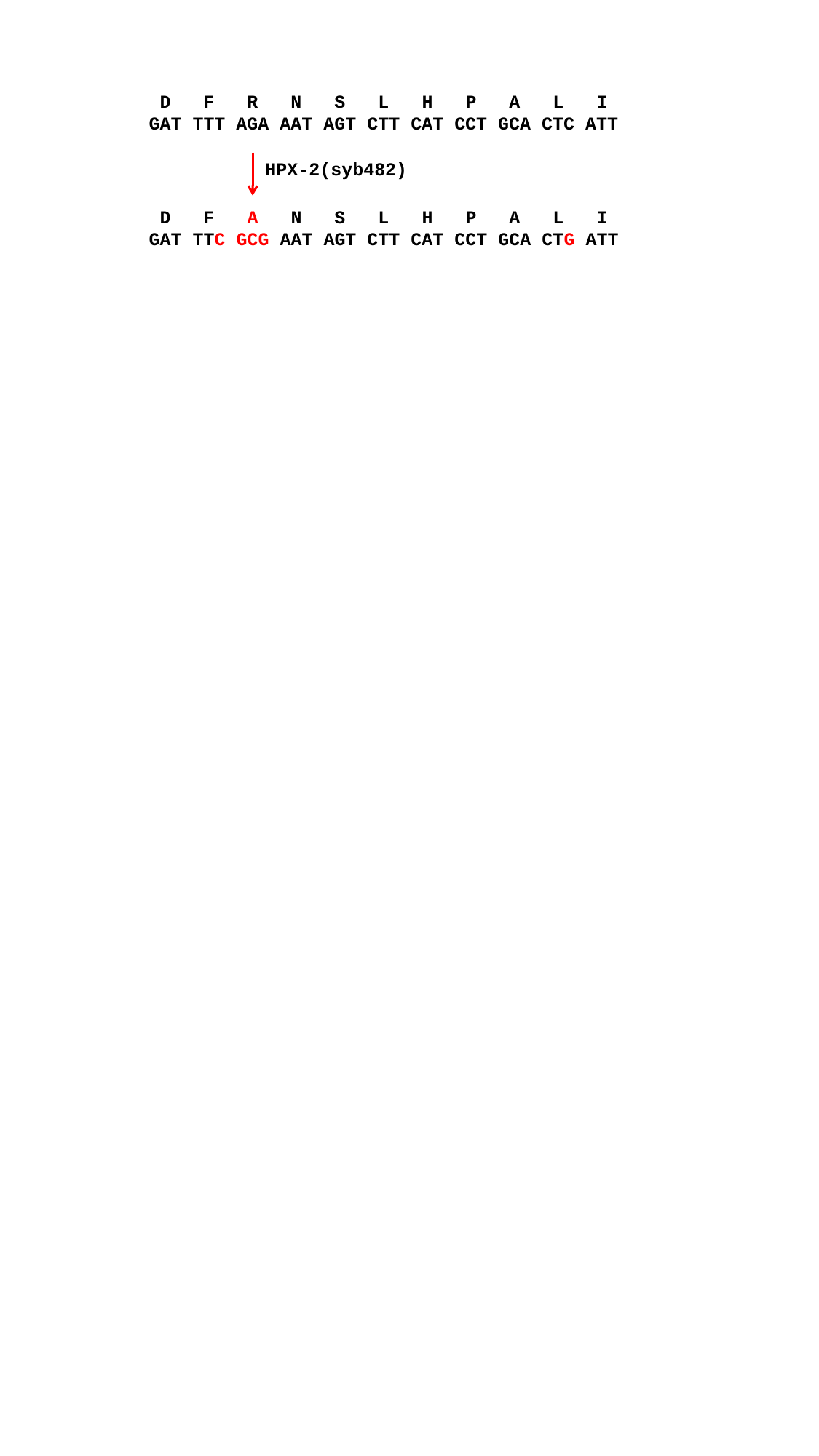

Figure S6
 D F R N S L H P A L I
GAT TTT AGA AAT AGT CTT CAT CCT GCA CTC ATT
HPX-2(syb482)
 D F A N S L H P A L I
GAT TTC GCG AAT AGT CTT CAT CCT GCA CTG ATT
